# Antidepressant Sertraline Is a Broad-Spectrum Inhibitor of Enteroviruses Targeting Viral Entry through Neutralization of Endolysosomal Acidification

**DOI:** 10.3390/v14010109

**Published:** 2022-01-08

**Authors:** Kuan-Chi Tseng, Bang-Yan Hsu, Pin Ling, Wen-Wen Lu, Cheng-Wen Lin, Szu-Hao Kung

**Affiliations:** 1 Department of Biotechnology and Laboratory Science in Medicine, National Yang Ming Chiao Tung University, Taipei 112304, Taiwan; kenchi@ym.edu.tw (K.-C.T.); kazw95001@yahoo.com.tw (B.-Y.H.); 2 Department of Microbiology and Immunology, National Cheng Kung University, Tainan 70101, Taiwan; lingpin@mail.ncku.edu.tw; 3 Department of Clinical Pathology, Cheng Hsin General Hospital, Taipei 11220, Taiwan; ch3538@chgh.org.tw; 4 Department of Medical Laboratory Science and Biotechnology, China Medical University, Taichung 40402, Taiwan; cwlin@mail.cmu.edu.tw

**Keywords:** enterovirus, antidepressant sertraline, drug repurposing, viral entry, host-cell targets, broad-spectrum antiviral

## Abstract

Enterovirus 71 (EV71) is an etiological agent of hand foot and mouth disease and can also cause neurological complications in young children. However, there are no approved drugs as of yet to treat EV71 infections. In this study, we conducted antiviral drug screening by using a Food and Drug Administration (FDA)-approved drug library. We identified five drugs that showed dose-dependent inhibition of viral replication. Sertraline was further characterized because it exhibited the most potent antiviral activity with the highest selectivity index among the five hits. The antiviral activity of sertraline was noted for other EV serotypes. The drug’s antiviral effect is not likely associated with its approved indications as an antidepressant and its mode-of-action as a selective serotonin reuptake inhibitor. The time-of-addition assay revealed that sertraline inhibited an EV71 infection at the entry stage. We also showed that sertraline partitioned into acidic compartments, such as endolysosomes, to neutralize the low pH levels. In agreement with the findings, the antiviral effect of sertraline could be greatly relieved by exposing virus-infected cells to extracellular low-pH culture media. Ultimately, we have identified a use for an FDA-approved antidepressant in broad-spectrum EV inhibition by blocking viral entry through the alkalization of the endolysosomal route.

## 1. Introduction

Enteroviruses (EVs) are non-enveloped, single-stranded RNA viruses belonging to the *Picornaviridae* family. The genus *Enterovirus* comprises many human pathogens, including poliovirus, coxsackie A and B viruses, echoviruses, numbered enteroviruses, and rhinoviruses. Diverse EVs can cause a broad range of diseases including hand, foot, and mouth disease (HFMD), encephalitis, aseptic meningitis, myocarditis and various respiratory diseases. Although most EV infections are mild, the symptoms can be severe in the young children and infants [1,2]. Among EVs, EV71 (or EV-A71) has emerged as a severe public health threat because it has caused major outbreaks of HFMD with a considerable number of affected individuals progressing to severe neurological complications and even death, mainly in the Asia-Pacific region [1,3]. Although many potent EV inhibitors have exhibited efficacy in cell cultures and animal models, effective antiviral agents for the clinical treatment of EV71 infections have not yet been approved [4,5]. The clinical course for the control of EV71 infections is currently limited to symptomatic treatment. Moreover, broad-spectrum drugs should be urgently developed because no single EV serotype is exclusively associated with any particular manifestations.

The EV71 life cycle begins from its attachment through the binding of the virus capsid protein to cell receptors [6,7]. This is followed by the entry of the virus mainly through clathrin-mediated endocytosis [8]; however, multiple pathways may be used by EV71 to enter diverse host cells [9,10,11,12]. Subsequently, a series of conformational changes can occur at low pH, followed by the release of viral RNA in the cell cytoplasm [8,10,11]. The positive-sense virus genome acts as an mRNA and is directly translated into a polyprotein through a mechanism of internal ribosome binding. Two viral proteases, 2A (2A^pro^) and 3C (3C^pro^), process the polyprotein, thus generating structural and nonstructural proteins. The RNA genome replication is performed by RNA dependent RNA- polymerase, termed 3D. Finally, the structural proteins and the progeny viral genome is encapsidated to form new virions that are released from the cell through lytic and non-lytic mechanisms [4,13].

We have developed a cell-based biosensor, HeLa-G2AwtR, which exhibits the conversion of fluorescence resonance energy transfer (FRET) upon an EV71 infection [14]. Because the FRET levels demonstrated by the HeLa-G2AwtR biosensor were reversely correlated with EV71 replication, this biosensor was used as a drug screening platform to identify anti-EV71 compounds [15]. In this study, we used the HeLa-G2AwtR biosensor for the drug repurposing screen of a library containing 774 FDA-approved drugs. The results revealed that five hits could inhibit EV71 infection in a dose-dependent manner. Sertraline was further characterized because it emerged as the most potent with the least cytotoxicity among the five hits. The antiviral activity of sertraline was not correlated with its well-known activity of selective serotonin reuptake inhibition that is clinically used for the treatment of major depression and obsessive compulsive disorders [16,17]. We found that Sertraline exerted broad-spectrum anti-EV activities independent of cytotoxicity. Our findings indicated that sertraline targeted viral entry possibly through neutralizing low-pH intracellular compartments and interfering with the endo-lysosomal entry process. To our best knowledge, this is the first study that addresses sertraline as an effective EV entry blocker and its associated mechanism-of-action.

## 2. Materials and Methods

### 2.1. Cells and Viruses

Rhabdomyosarcoma (RD) (ATCC, CCL-136), HeLa (ATCC, CCL-2), and HeLa-G2AwtR cells [14] were cultured in minimum essential medium (MEM) (Gibco-BRL Inc.) supplemented with 10% fetal bovine serum (FBS). EV stocks including EV71 (strain BrCr), coxsackievirus A16 (CVA16), CVB1 CVB2, echovirus serotype 9 (Echo9), and Echo30 [18] were propagated in RD cell culture with MEM-2, and titrated on a RD cell monolayer by using a plaque assay [19].

### 2.2. Chemicals and Drug Library

Sertraline (79559-97-0), fluvoxamine (61718-82-9), paroxetine (110429-35-1), chlorpromazine (69-09-0), chloroquine (50-63-5) and ribavirin (36791-040-5) were purchased from Merck Inc. (Kenilworth, NJ, USA). A total of 774 compounds listed in the FDA (v.2.0) Approved Drug Library (BML-2843-0100) were purchase from Enzo Life Sciences Inc. (Farmingdale, NY, USA) and provided by Dr. Yeou-Guang Tsay (Supplementary Table S1). All the drugs were dissolved in dimethyl sulfoxide (DMSO), and the final concentration of DMSO in the culture medium did not exceed 0.05%, a concentration tolerated by all cell lines tested. All drug-free control contained 0.05% DMSO.

### 2.3. Drug Screening Format

We used the screening protocol reported in a previous study [15] with some modifications. Briefly, HeLa-G2AwtR cells in black 96-well dishes (SPL Life Sciences, Pocheon, Korea) were pretreated with drugs from the library at a concentration of 5 μM for 1 h, and the original medium was replaced with a medium containing EV71 stock at a multiplicity of infection (MOI) of 1 and the corresponding test drugs at 5 μM for 12 h. Drug-free, infected cells and drug-free, mock-infected cells, each in triplicate in a dish, were used as the infected control and uninfected control, respectively. Ribavirin, a known EV replication inhibitor [20], served as the positive control. The fluorescence intensities of the treated HeLa-G2AwtR cells were measured using a fluorometer (Fluoroskan Ascent type 374; Labsystems, Vantaa, Finland) with an excitation wavelength of 390 nm. The emission wavelengths for the fluorophore donor (green fluorescent protein 2, GFP^2^) and acceptor (red fluorescent protein, DsRed2) were 510 and 590 nm, respectively. The averages of FRET ratios for the infected HeLa-G2AwtR and mock-infected cells were arbitrarily set as 0% and 100% inhibition rates, respectively. We arbitrarily classified a test drug as a hit when the FRET ratio increased by >50% in the window between the infected and uninfected controls.

### 2.4. Cell Viability Assay

The drug concentrations tested were 0.1, 0.3, 1, 3, and 10 μM. Cell viability was determined after a 12 h treatment using the CellTiter 96 AQ_ueous_ Cell Proliferation Assay (Promega, Madison, WI, USA) as previously described [18]. The 50% cytotoxicity concentration (CC_50_) was calculated using GraphPad Prism5 (GraphPad Software, San Diego, CA, USA).

### 2.5. Immunofluorescence Assay

RD cells grown in 24-well dishes were fixed with 4% paraformaldehyde and penetrated using 0.2% Triton X-100. The following antibodies (Abs) were used to probe EV serotypes: mouse anti-EV71 Ab (1:1000; MAB979, Merck Inc., Kenilworth, NJ, USA) for EV71 and CVA16; mouse anti-coxsackie virus B blend Ab (1:1000; MAB9410, Merck Inc., Kenilworth, NJ, USA) for CVB1 and CVB2; and mouse anti-echovirus blend Ab (1:1000; MAB9670, Merck Inc., Kenilworth, NJ, USA) for Echo9 and Echo30. A fluorescein isothiocyanate (FITC)–conjugated goat antimouse Ab (1:100; Jackson) was used as a secondary Ab. The nuclei were counterstained with 4′, 6-diamidino-2-phenylindole (DAPI; 0.1 µg/mL; Merck Inc., Kenilworth, NJ, USA). The cells were viewed under a fluorescent microscope (Leica DM6000B) equipped with both FITC and UV filters. EV antigen–positive cells and DAPI-positive cells from each field (at least four fields from each experiments) were counted and analyzed using the associated MetaMorph software (Nashville, TN, USA).

### 2.6. Western Blot

A Western blot analysis was conducted following the procedure used in a previous study [18]. The abs used to probe EV71 antigen were a mouse anti-EV71 Ab (1:1000; MAB979, Merck Inc., Kenilworth, NJ, USA) as a primary Ab and a horseradish peroxidase (HRP)-conjugated goat anti-mouse polyclonal Ab (1:1000, sc-2060, Santa Cruz Biotechnology, Santa Cruz, CA, USA) as a secondary Ab. The α tubulin was a loading control probed by a rabbit anti-α tubulin primary Ab (1:1000, GTX112141, Gene Tex) and a goat anti-rabbit HRP-conjugated secondary Ab (1:3000, sc-2004, Santa Cruz Biotechnology, Santa Cruz, CA, USA). Proteins were detected using an enhanced chemiluminescence (ECL) Western blot kit (Perkin Elmer Inc., Norwalk, CT, USA).

### 2.7. RNA Extraction and RT-qPCR

RNA preparation and RT-qPCR followed the protocol previously described [18]. The total cellular and viral RNA was extracted using a TRIzol reagent (ThermoFisher Inc., Carlsbad, CA, USA). The reverse transcription (RT) reaction and real-time PCR were performed using an AMV Reverse Transcriptase XL (Takara, Kusatsu, Japan) and the FastStart Universal SYBR Green Master kit (Roche Applied Science, Penzberg, Germany), respectively, according to the manufacturer’s instructions. PCR primer pairs for the VP1 region of the EV71 (BrCr strain) genome were: forward primer: 5′-AGTATGATTGAGACTCGGTG-3′ and reverse primer 5′-GCGACAAAAGTGAACTCTGC-3′ [21]. PCR primers used for the human β-actin were: forward primer: 5′-AGCCTCGCCTTTGCCGA-3′ and reverse primer 5′-CTGGTGCCTGGGGCG-3′ [22]. Relative viral RNA level (%) was calculated using the viral cDNA level normalized against that of the human β-actin, and compared with that of the DMSO control, as described previously [22].

### 2.8. The Binding and Internalization Assay

For the binding assay, RD cells pretreated with were pre-chilled at 0 °C for 10 min. Culture medium was removed and cells were inoculated with EV71 stock at 5 MOI in the presence of sertraline at 5-, 10-μM or left untreated at 0 °C for 1 h. Unbound virions were removed by washing three times with ice-cold PBS. For the internalization assay, unbound virus was removed after binding and replaced with pre-warm medium containing respective concentrations of sertraline, and incubated at 37 °C for 1 h. Cells were trypsinized (0.25% trypsin, Gibco Inc., Brooklyn, NY, USA) at 37 °C for 3 min to remove surface-bound viruses.

### 2.9. Lysotracker-Flow Cytometry

This experiment followed the previous studies [23,24] with some modifications. RD cells were pretreated with indicated drugs at 5 and 10 μM for 2 h at 37 °C and replaced with LysoTracker DND-99 (150 nM, cat. no. L7528, ThermoFisher Inc., Carlsbad, CA USA) containing the same concentration of drug at 37 °C for 1 h. Subsequently, the cells were washed in phosphate-buffered saline (PBS) for three times, trypsinized, centrifuged, and resuspended in 500 μL of sorting buffer and 500 μL of 4% paraformaldehyde according to the manufacturer’s instructions. The resuspended cells were shaken for 30 min, and their fluorescence was measured using a Beckman Coulter CytoFLEX S at an excitation/emission wavelength of 561/585 ± 42 nm, and data were analyzed using CytExpert software.

### 2.10. The Low-pH Exposure Assay

We used a protocol reported in a previous study [25] with some modifications. The cells were pretreated with a medium containing 10 μM sertraline for 1 h at 37 °C. Following pretreatment, the cells were infected with EV71 at an MOI of 0.1 in the presence of 10 μM sertraline at 0 °C. The inoculum was removed, and unbound virus was washed away with ice-cold PBS. The cells were incubated for 1 h at 37 °C in the presence of sertraline. The supernatant was removed, and media with different pH levels (7.4, 6.5, 5.5, and 5.0) containing 10 μM sertraline were added for 10 min. The media were removed, and cells were incubated in medium (pH 7.4) containing 10 μM sertraline at 37 °C for 6 h and 12 h for measurement of relative levels of the viral protein and the viral titer, respectively.

## 3. Results

### 3.1. Drug Screening

**Figure 1:**
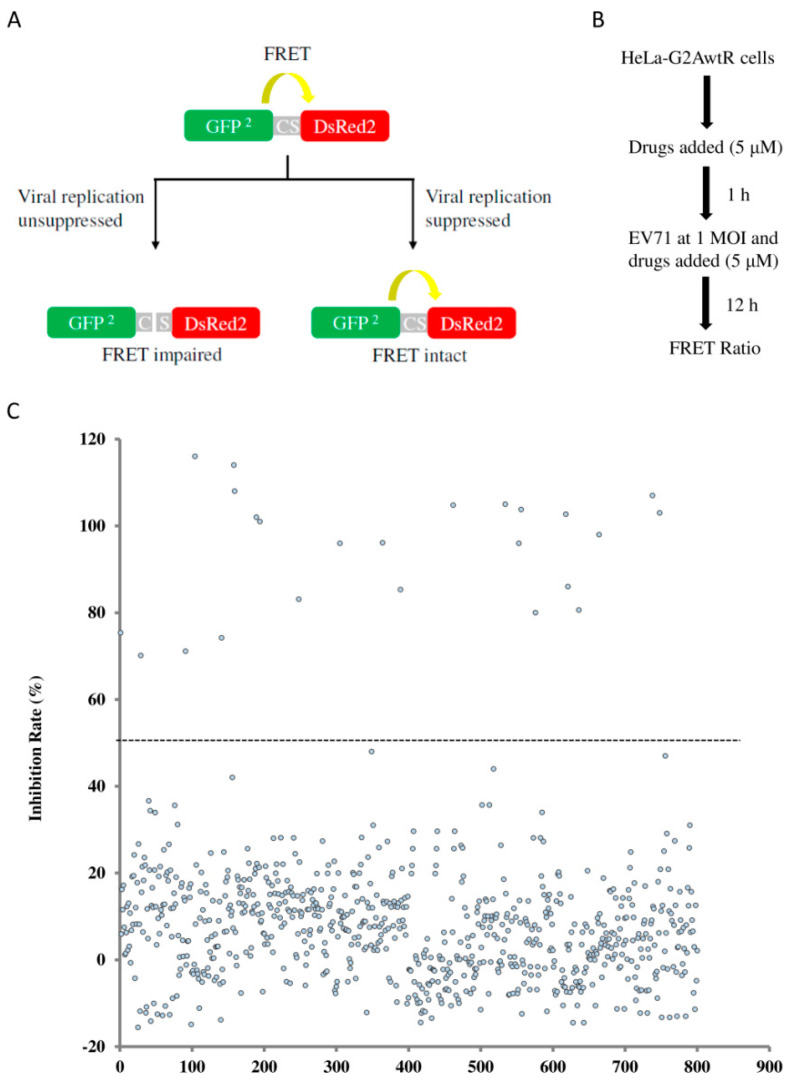
Fluorescence resonance energy transfer (FRET)-based biosensor for the drug screening of EV71 inhibitors. (**A**) Diagram illustrating the principle of FRET-based drug screening by using HeLa-G2AwtR cells where the green fluorescence protein (GFP^2^)–red fluorescence protein (DsRed2) tandem fluorophores with the cleavage site (CS) of virus 2A protease in between is stably expressed. FRET occurs due to the close proximity of the fluorophore pair. FRET is reduced upon the viral protease cleavage in the context of EV71 replication, whereas FRET remains intact when viral replication is significantly blocked. (**B**) Flowchart of the FRET-based biosensor for drug screening with the BML-2843-0100 drug library. (**C**) Scatter plot of the inhibition rate resulted from the action of test drugs. A total of 24 drugs from the screen yielded an inhibition rate of >50%, as indicated. The average FRET ratios from EV71-infected HeLa-G2AwtR cells and mock-infected cells were arbitrarily set as 0% and 100% inhibition rates, respectively.

To screen the anti-EV71 drugs, we used the HeLa-G2AwtR biosensor cells that exhibit reduced FRETs in response to viral multiplication. This was achieved by the stable expression of a recombinant substrate composed of the green fluorescent protein 2 (GFP^2^) and the red fluorescent protein (DsRed2) linked by a viral 2A^pro^ cleavage motif. FRET from the biosensor cells was pronounced owing to the close proximity of the FRET pair until EV71 infection that disrupted the recombinant substrate through the substrate cleavage by the viral 2A^pro^ [15] (Figure 1A). A library consisting of 774 FDA-approved drugs was used for a repurposing screen (Supplementary Table S1). The HeLa-G2AwtR cells were incubated with each test drug at a concentration of 5 μM prior to viral inoculation, and the compounds remained in the medium throughout the duration of the infection for 12 h, a period for an infectious cycle of EV71. The FRET ratio of each well was calculated by measuring emission fluorescence from the acceptor molecule divided by that from the donor molecule (Figure 1B). The Z’-factor that reflects statistical stability was calculated; Z’-factors monitored in each screening plate were in the range of 0.54 to 0.63, indicating a robust assay performance. This primary screen captured 24 hits that caused a >50% inhibition rate (Figure 1C). Among them, idarubicin, doxorubicin, daunorubicin, epirubicin, mitoxantrone and gemcitabine are known inhibitors of EV replication [15,18,20,26], thus, they served as internal controls for the sensitivity of the screen. The primary hits were used for secondary screening based on <20% cell cytotoxicity at 10 μM and dose-dependent inhibition. Fingolimod, hexachlorophene, mefloquine, nibivolol and sertraline scored as novel hits from the secondary screen; their approved indications and mode-of-action have been reported [16,17,27,28,29,30] (Table 1).

### 3.2. The Antiviral Potency and Spectrum

**Figure 2:**
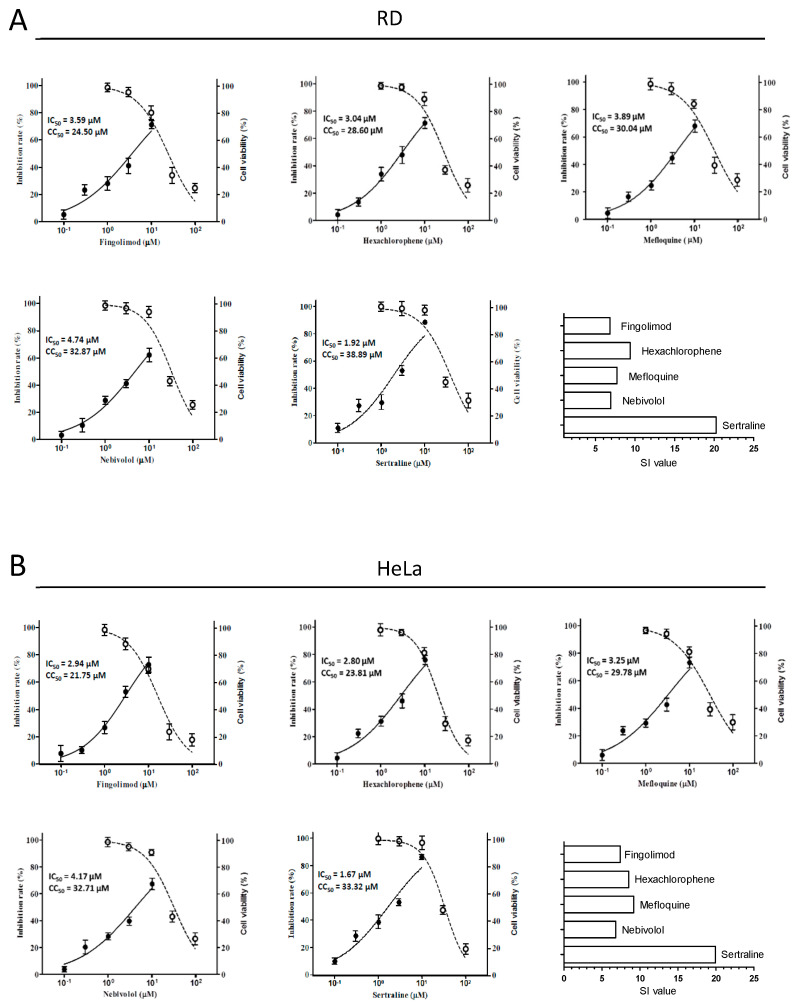
Dose–response relationships of novel anti-EV71 hits identified from the screen. (**A**) RD and (**B**) HeLa cells were pretreated for 1 h at increasing concentrations (0.1, 0.3, 1, 3, and 10 μM) of the indicated drugs and then infected with EV71 stocks at a multiplicity of infection (MOI) of 0.1, with test drugs maintained throughout the infection. At 12 post-infection (p.i.), the cells were analyzed using an immunofluorescence assay (IFA). For each condition, the percentage of infection was calculated as the ratio of the number of infected cells stained for viral VP1 to the number of cells stained with DAPI. Drug cytotoxicity was determined by treating HeLa or RD cells with increasing concentrations of the indicated drugs (1, 3, 10, 30, and 100 μM) for 12 h. Cell viability was measured using a cell proliferation assay and expressed as the percentage of drug-free cells. IC_50_ and CC_50_ values were calculated using GraphPad Prism5 software. The solid circle and empty circle represented the inhibition rate (%) and cell viability (%), respectively. The Selective Index (SI) value was calculated by value of CC_50_ divided by that of IC_50_. Data represented the means of triplicated experiments and the SEM. Drug-free wells contained 0.05% DMSO.

Next, the anti-EV71 potency of the five compounds was examined using an immunofluorescence assay (IFA). In the IFA, we used the indicated concentrations of the compounds in both the RD and HeLa cells. For all cases, dose–response inhibition was observed, and the 50% inhibition concentration (IC_50_) was calculated for each drug. In addition, a cell viability assay was performed to examine the 50% cytotoxicity concentration (CC_50_) of each drug, and the selectivity index (SI = CC_50_/IC_50_) for each drug was obtained (Table 1 and Figure 2). These data indicated that these five drugs exerted antiviral effects with IC_50_s, ranging from 1.92 to 4.74 μM and from 1.67 to 4.17 μM in the RD and HeLa cells, respectively, and that their effects were independent of cytotoxicity.

**Figure 3:**
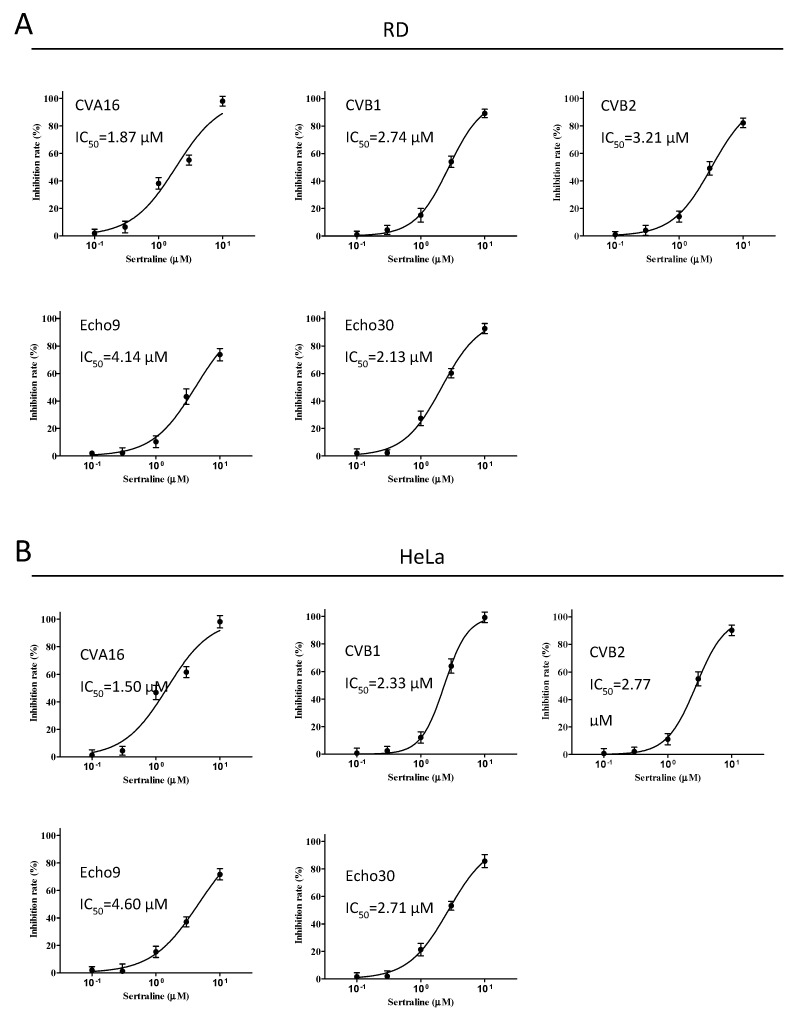
Antiviral activity of sertraline against five other EV serotypes. (**A**) RD and (**B**) HeLa cells were pretreated for 1 h at increasing concentrations (0.1, 0.3, 1, 3, and 10 μM) of sertraline and subsequently infected with CVA16, CVB1, CVB2, Echo9, and Echo30 stocks at an MOI of 0.1 for 12 h, with the corresponding concentration of sertraline maintained throughout the infection. The IFA was conducted using the aforementioned protocol. Primary antibodies (Abs) were the mouse anti-EV71 Ab for EV71 and CVA16, mouse anti-coxsackie virus B blend Ab for CVB1 and CVB2, and mouse anti-echovirus blend Ab for Echo9 and Echo30. The secondary Ab was the FITC-conjugated goat anti-mouse Ab. The inhibition rate (%) was measured as described in Figure 2. Triplicated experiments were conducted with the mean and the SEM shown. Drug-free wells contained 0.05% DMSO.

Sertraline (Supplementary Figure S1) was chosen for further characterization because this drug exhibited the strongest inhibition with relatively high selectivity indexes (Sis~20). We examined the antiviral spectrum of sertraline against a panel of representative EV serotypes, namely CVA16, CVB1, CVB2, Echo9, and Echo30. In all the species, sertraline exerted antiviral effects albeit to various extents, with IC_50_ values ranging from 1.87 to 4.14 μM and from 1.50 to 4.60 μM in the RD and HeLa cells, respectively (Figure 3).

### 3.3. Sertraline Targeted the Viral Entry

The antidepressant sertraline is a selective serotonin reuptake inhibitor (SSRI) and is unrelated to any of the clinically used antivirals. Mechanically, this class of drug acts by increasing the local concentration of serotonin at synapses and in extracellular spaces by targeting the serotonin transporter (SERT), thus blocking serotonin transport back to presynaptic neurons [16,17]. To determine whether the antiviral activity of sertraline is associated with the approved indications, we first used the Human Protein Atlas data set to determine the gene expression levels of SERTs in RD and HeLa cells. Although SERTs are abundantly expressed in brain tissues, they are minimally expressed in RD cells (striated muscle cells) and HeLa cells (Supplementary Section S2). These findings indicated that the antiviral activity of sertraline might not be associated with its selective serotonin reuptake inhibition activity.

**Figure 4:**
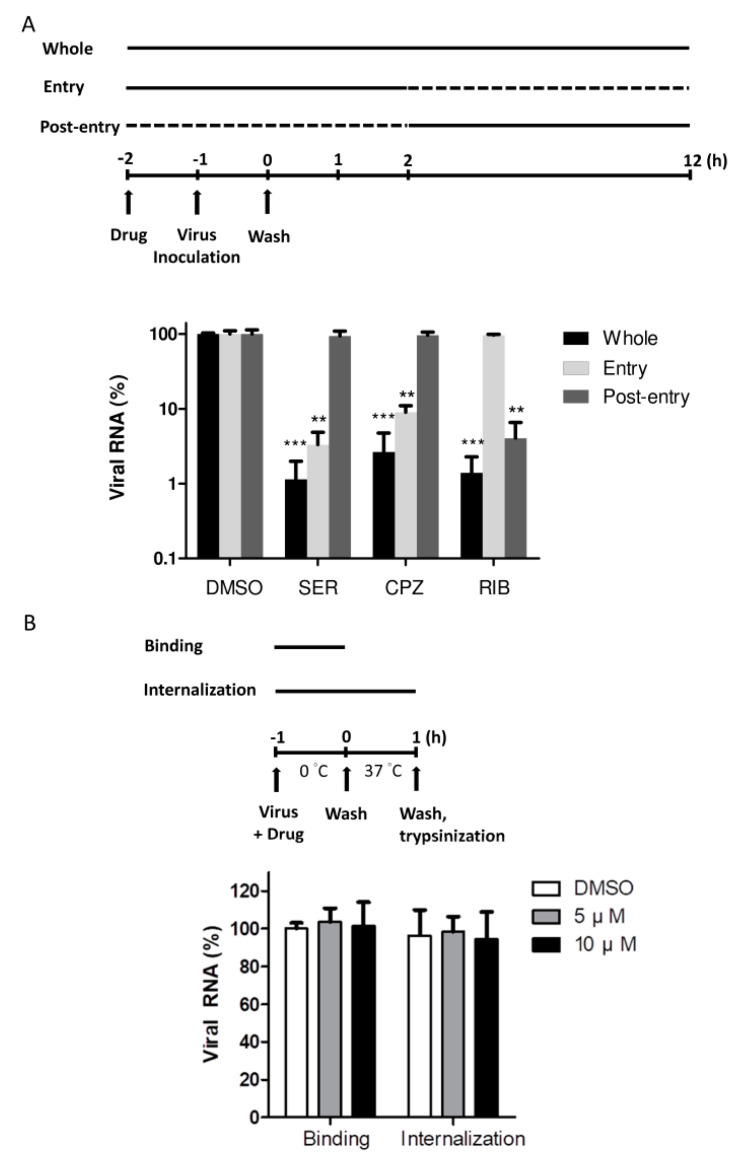
Timing of EV71 inhibition by sertraline. (**A**) Time-of-addition assay with the schematic representation. Each compound was added to RD cells at the indicated times relative to viral infection at an MOI of 0.1 at 37 °C, with the initiation (−1 h) and completion (0 h) of viral adsorption indicated. Solid and dashed lines indicate the periods with and without compound treatment, respectively. Whole: −2 to 12 h; entry: −2 to 2 h; post-entry: 2–12 h. Sertraline (SER), 10 μM; chlorpromazine (CPZ), 20 μM; ribavirin (RIB), 100 μM. (**B**) The binding and internalization assay with the schematics. EV71 at an MOI of 5 was allowed to bind to the cells on ice for 1 h in the presence of 5 or 10 μM of sertraline or without drug treatment. For the binding assay, unbound virus was extensively washed off. For the internalization assay, the cells were warmed to 37 °C for 1 h after incubation on ice, allowing viruses to enter cells. Cell surface–bound viruses were removed using trypsin. Quantitative reverse transcription polymerase chain reaction (RT-qPCR) was conducted using total RNA prepared from each condition in (**A**,**B**), and shown as relative levels to drug-free control containing 0.05% DMSO. These data were from three independent experiments. ** *p* < 0.01; *** *p* < 0.001.

To identify the stage of viral infection inhibited by sertraline, we performed a time-of-addition experiment in a single infectious cycle for 12 h. We added 10 μM of sertraline to RD cells at the following times: (a) 1 h before and during the 1 h virus adsorption step and maintained throughout the 12 h infection period, (b) 1 h before and during the 1 h virus adsorption step and for an additional 2 h and then removed (entry step), or (c) 2 h after the adsorption step and maintained for the remaining 10 h of infection (post-entry). Chlorpromazine (CPZ), a cationic amphiphilic reagent that blocks the assembly of the clathrin adaptor protein AP2, is known to inhibit entry of EV71 in RD cells through clathrin-dependent endocytosis [8,10]; thus, it was used as a control as entry inhibitor. Ribavirin is a nucleoside analog that targets EV71 genome replication so it served as a control for the post-entry step [20]. For each condition, the relative levels of viral RNA were analyzed through quantitative reverse transcription polymerase chain reaction (RT-qPCR; Figure 4A). We showed that treatment of sertraline resulted in a profound reduction in viral RNA levels at the entry step with a negligible effect on the post-entry step.

To determine the precise point at which EV71 viral entry was affected by sertraline, we investigated the effects of sertraline on virus binding and internalization. For the virus-binding assay, the cells were incubated with the virus at 0 °C for 1 h with or without sertraline. For the virus internalization assay, the temperature was increased to 37 °C for 1 h (after virus binding) to allow for the internalization of bound virus particles, followed by trypsinization to remove uninternalized surface virus particles [31,32]. For each condition, viral RNA levels were measured through RT-qPCR. An undetectable difference was observed between the drug-treated group and drug-free control, indicating that neither virus binding nor virus internalization was targeted by sertraline (Figure 4B).

### 3.4. Sertraline Neutralized the pH-Dependent Virus Entry

**Figure 5:**
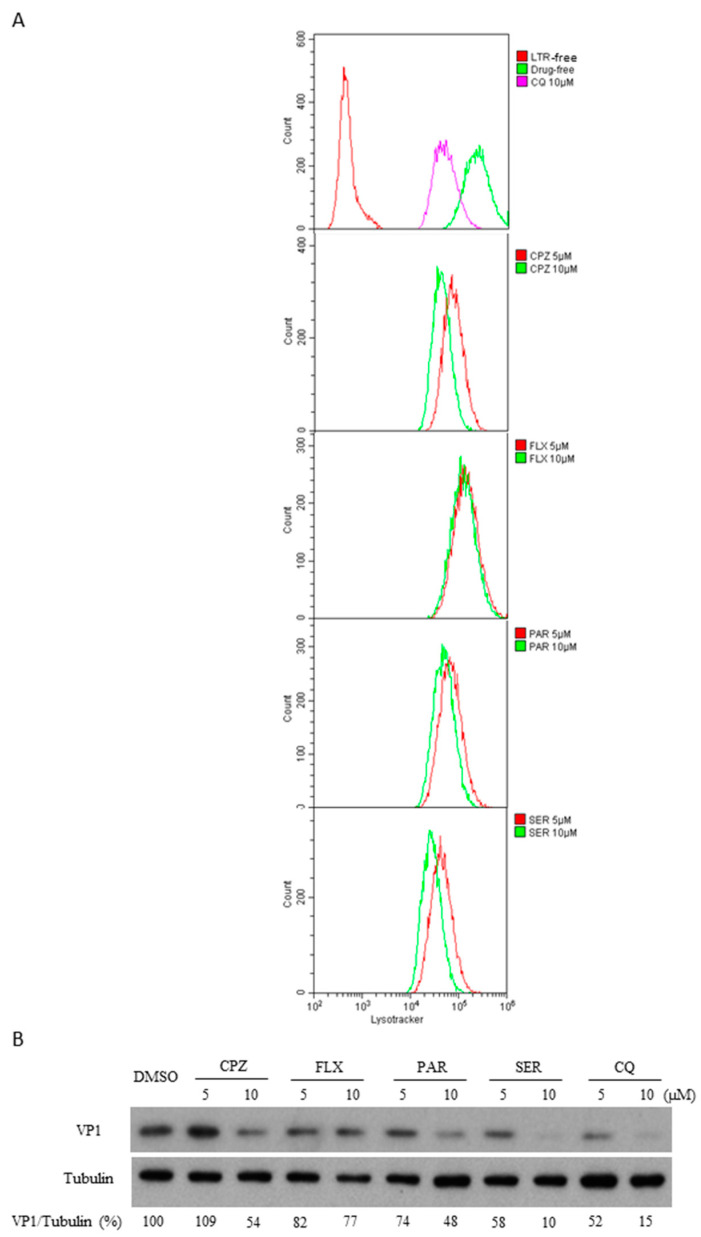
The alkalizing effect of selective serotonin reuptake inhibitors tested was associated with the antiviral potency. (**A**) RD cells were treated with chloropromazine (CPZ), fluvoxamine (FLX), paroxetine (PAR), sertraline (SER), and chloroquine (CQ) at 5 and 10 μM for 2 h. The cells were labeled with 150 nM Lysotracker (LTR) for 1 h. Flow cytometry was performed using the indicated mean fluorescence intensity value. Histogram overlays of LTR-free control (in the top panel) and LTR-labeled cells in the absence or presence of indicated drugs are shown. (**B**) RD cells were pretreated with the indicated drugs at 5 or 10 μM for 1 h. Culture media were replaced with EV71 stock at an MOI of 1 and the corresponding drugs of the same concentrations for 1 h. Culture media were removed and replaced with media containing the corresponding drugs of the same concentrations for 6 h. Cell lysates were prepared and subjected to Western blot analysis using the mouse anti-EV71 VP1 antibody, using the antitubulin antibody as the internal control. The percentage shown below each lane represents the intensity of viral VP1 relative to that of tubulin.

Next, we investigated endosomal trafficking, a step following the viral internalization step at the viral entry stage. Sertraline shows a structural and physicochemical property characteristic of lysomotropic cationic amphiphilic drugs (CADs) (Supplementary Figure S1). CADs contain a hydrophobic aromatic ring and segregated hydrophilic segments with an ionizable amine functional group. Owing to their amphiphilic nature, CADs tend to permeate the membrane, and they undergo protonation and are trapped inside acidic intracellular compartments such as late endosomes and lysosomes. This process can result in alkalization and the consequent physiological impairment of the endolysosomal pathway crucial for EV entry [33,34,35]. We examined whether sertraline treatment caused the alkalization of acidic cellular compartments labeled by the LysoTracker dye that emits red fluorescence only when the dye has entered an acidic milieu such as endolysosomes [23,34]. Two other known SSRIs, fluvoxamine and paroxetine, were used for comparison (Supplementary Figure S1) [16]. Two CADs, chlorpromazine and chloroquine, were included because chlorpromazine can target CME and chloroquine is a known inhibitor of endosomal acidification; both the drugs inhibit EVs independent of the SSRI activity [8,36]. The results of flow cytometry analysis revealed that among the three tested SSRIs, sertraline exerted the strongest alkalization effect, followed by paroxetine and fluvoxamine, according to the curve-shifting profile (Figure 5A). Next, these drugs were used to examine antiviral activity against EV71 infections. Sertraline displayed the strongest inhibitory effect, followed by paroxetine, whereas fluvoxamine exhibited the weakest antiviral activity (Figure 5B). These data indicated that the ability of endosomal alkalization is positively correlated with antiviral activity, which is likely independent of SSRI activity.

**Figure 6:**
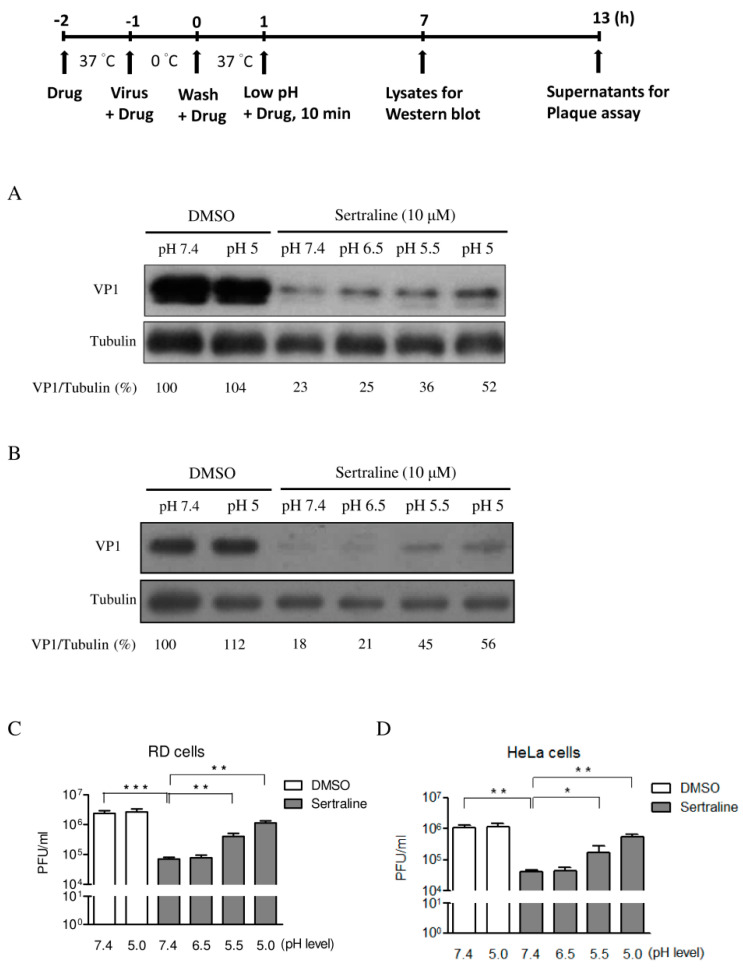
Exposure of the cells to low pH alleviated the antiviral activity of sertraline. The low-pH exposure assay with the schematic representation. (**A**,**C**) RD and (**B**,**D**) HeLa cells were pretreated with 10 μM sertraline for 1 h. EV71 was used to inoculate the pretreated cells at an MOI of 1 in the presence of 10 μM sertraline on ice for 1 h. The supernatant containing unbound virus was removed, and the cells were incubated with medium containing 10 μM sertraline at 37 °C for 1 h. The cells were incubated with media with different pH values (7.4, 6.5, 5.5, and 5.0) that contained 10 μM sertraline for 10 min and then replaced with 10 μM sertraline-containing medium (pH 7.4). (**A**,**B**) At 6 h after the addition of the media with the different pH values, levels of viral VP1 protein and tubulin (the internal control) were measured by Western blot. The percentage shown below each lane represents the intensity of viral VP1 relative to that of tubulin. (**C**,**D**) At 12 h after the addition of the media with the different pH values, viral titers from the supernatants were titrated by plaque assay. Triplicated experiments were conducted (* *p* < 0.05; ** *p* < 0.01; *** *p* < 0.001).

As sertraline raised the pH level in the acidic intracellular compartments, we posited that the antiviral effect of sertraline could result from the blockade of endosomal acidification. For this, we exposed RD and HeLa cells to low extracellular pH values after virus binding. The cells were pretreated with 10 μM sertraline and then inoculated with EV71 stock at 1 MOI in the presence of 10 μM sertraline on ice. At 1 h post-infection, the inoculum was removed and unbound viruses were washed off. The viruses were allowed to enter into the cells by incubating them in the presence of 10 μM sertraline at 37 °C for 1 h. Subsequently, the cells were incubated for 10 min in media at different pH values (7.4, 6.5, 5.5, and 5.0) containing 10 μM sertraline. The cells were incubated for another 6 h or 12 h in sertraline-containing medium (pH 7.4). The cell lysates were prepared for the Western blot analysis of the levels of viral protein at 6 h p.i. (Figure 6A,B). We found that viral replication at the levels of viral protein could be markedly rescued by the low-pH shocks (pH 5.5 and 5) in a pH-dependent manner in both distinct cell types. In addition, the supernatants were collected for the titration of the viral yields at 12 h p.i. (Figure 6C,D). The viral titers were reduced to 2.9% and 3.9% for the RD and HeLa cells, respectively, relative to the DMSO-pH 7.4 controls upon the treatment of 10 μM sertraline (pH 7.4). The alleviation of sertraline blockade was even more pronounced in that the exposure of pH 5.5 and 5 media resulted in 5.7 and 16 times more viral titers, respectively, than that of the sertraline-treated pH 7.4 control in RD cells. A similar trend was found with 4 and 13.4 times more viral titers after the pH 5.5 and 5 medium exposure, respectively, than that of the sertraline-treated pH 7.4 control in HeLa cells (Figure 6D). In marked contrast, exposure of the cells to media at pH 6.5 did not result in significant recovery of viral replication at the levels of viral protein and viral yield, consistent with the notion that the early endosomes (at pH 6.0–6.5) play a minor role in the viral entry [8]. This result agrees with our previous finding that sertraline treatment caused the alkalization of the cellular acid compartment (Figure 5A). Attempts to optimize the exposure time to extracellular acid media revealed that a longer exposure time (≥20 min) was cytotoxic, whereas a shorter exposure time (5 min) resulted in lesser efficiency in alleviation of the inhibitory effect by sertraline (data not shown). Together, the results indicated that sertraline likely targeted a low pH-dependent step of the viral entry process.

## 4. Discussion

In this study, we searched for new candidate drugs against EV71 infections by screening a repurposing library of 774 drugs using a FRET-based biosensor. Among the five hits, sertraline exerted the most potent antiviral activity with the highest SI value. The antiviral activity of sertraline is not likely associated with its mode of action as an SSRI for approved indications because (A) the sertraline transporter targeted by SSRIs is not expressed in the RD and HeLa cells employed in this study and (B) two other SSRIs we tested, paroxetine and fluvoxamine, exhibited much weaker antiviral activities. A serial mechanistic study showed that sertraline exerted the antiviral effect likely at the entry stage (Figure 4A), with the exclusion of the virus binding and internalization steps (Figure 4B). In addition, we observed that sertraline exhibited the lysomotropic feature that alkalizes cellular compartments, and compared with paroxetine and fluvoxamine, sertraline’s lysomotropism exhibits a stronger association with its antiviral activity. Moreover, the exposure of cells to the extracellular acidic medium markedly reversed the blockade of sertraline action in a pH-dependent manner in different cell types infected by EV71. Together, these data indicated the involvement of a mechanism through which sertraline neutralizes the pH level of the endolysosomal route, which is crucial for the entry of EVs.

Prior to this study, the antiviral activity of sertraline was reported on only against a few viral groups including filoviruses and coronaviruses [37,38]. Sertraline acts as a functional inhibitor of acid sphingomyelinase (ASM) that impairs the enzymatic activity of low-pH-dependent, lysosomal ASM [39,40]. In addition, ASM and its product ceramide play a crucial role in the entry of both filoviruses and coronaviruses [39,41,42]. It is therefore speculated that sertraline might intervene with the entry of both filoviruses and coronaviruses, at least partly, through the inactivation of ASM owing to its effect on the alkalization of the acid lysosomal milieu presented in this study. Moreover, the molecular modeling performed using the thermal shift assay indicated that sertraline directly binds with the envelope glycoprotein (GP) of Ebolavirus to destabilize GP conformation, thereby blocking the fusion between viral and endosomal membranes [43]. In addition, the main EV71 receptor scavenger receptor class B, member 2 (SCARB2) mediates viral attachment and uncoating. The SCARB2 is localized in the endolysosomal compartments and shuttles to the plasma membrane. Likewise, sertraline might target the SCARB2 in the acidic milieu along the endolysosomal pathway [7,11]. To the best of our knowledge, this is the first study to report that sertraline effectively blocks the entry of nonenveloped viruses, such as EVs, by acting through its lysomotropic feature. Whether lysosomal-resident proteins, such as ASM and SCARB2, are targets of sertraline merit further investigation.

Sertraline displays the physicochemical features of a lysosomotropic CAD that readily allows for the transmigration of membranes. Upon reaching acidic compartments such as late endosomes and lysosomes, the basic amine groups of sertraline undergo protonation, thus resulting in the lysosomal trapping of compounds and their subsequent accumulation in acidified compartments. Criteria for drugs with high possibility of lysosomotropic effects include lipophilicity, the protonation ability, and the tendency to accumulate in cells and induce phospholipidosis [34]. Therefore, SSRIs such as paroxetine and fluvoxamine used in this study exerted a lower lysosomotropic effect than did sertraline, at least partly owing to the variations in the factors above. While well-known lysosomotropic agents such as chloroquine exerted its inhibitory effect comparable that of sertraline (Figure 5B), other known lysosomotropic agent such as NH_4_Cl had much weaker antiviral effect in the range of several mM milimolar levels [8,11]. Moreover, in a previous study, the three SSRIs were tested on a mouse model for the pharmacological effects on attenuation of obsessive compulsive disorder behavior [44]. The drug potencies were in the order of paroxetine > sertraline > fluvoxamine, which is different from their lysomotropic effects presented in this study. The data further supported that the antiviral activity of sertraline is likely independent of its SSRI activity.

Sertraline is an FDA-approved drug with a long clinical history of use with approval for major depression and obsessive compulsive disorder. Drug repurposing of an approved drug can significantly reduce the time and resources required to advance a candidate drug into animal studies and clinical setting, because its safety and pharmacokinetic profiles have already been extensively evaluated. The reported maximum concentration of sertraline in human plasma is approximately 200 ng/mL (~654 nM) [45], which is lower than the in vitro IC_50_s we observed (Table 1 and Figure 2). Nevertheless, sertraline can concentrate in some organs including the brain, which is targeted by EV71 [2] by as much as 7.38 to 22-fold on average [46,47]; therefore, therapeutic concentrations may be achieved in some tissues. Moreover, EV71 acute infection would presumably require a shorter drug course than chronic treatment for depression-related disorders, thereby reducing drug loading. Another consideration is drug combination with sertraline as a component and other approved drugs to test for synergism, which may reduce the dosage and improve therapeutic selectivity [48,49].

The early stages of the virus lifecycle are ideal targets for preventing viral infection because, by this point, the virus has not yet multiplied. Our study suggested that the alkalization of acidic compartments in host cells is an effective strategy for reducing viral infection and that the lysosome is a viable target organelle for antiviral drug discovery. The pH-dependent, endolysosomal route is a common pathway shared by many viruses of distinct groups; therefore, the antiviral spectrum of sertraline merits further investigation. On the other hand, less-pH dependent EVs and viruses of other groups have been reported [50]. Underlying mechanisms other than acid milieu in the endolysosomal route have been proposed. These involve ionic conditions in the endolysosomes regulated by lysosomal ion channels such as the Ca^2+^ selective two-pore channels (TPCs) and transient receptor potential mucolipins (TRPMLs) [51]. The TPCs have been implicated in the viral entry of filoviruses and coronaviruses whereas the TRPMLs have been reported to promote the infectivity of influenza virus and Zika virus, both types of ion channels act through increased endosomal trafficking [52,53,54]. Whether the TPCs and TRPMLs mediate endosomal entry of EVs warrants investigation. Moreover, sertraline, as a host-directed antiviral agent, presents a higher bar to the development of drug resistance.

## Figures and Tables

**Table 1:** Antiviral Potency of the Hits, and Their Approved Indications and the Associated Modes of Action.

Drug Name	IC_50_/CC_50_ (μM)		Approved Indication	Mode of Action	Reference
	RD	HeLa			
Fingolimod	3.59/24.50	2.94/21.75	Anti-multiple sclerosis	Sphingosine-1-phosphate receptor modulator	[26]
Hexachorophene	3.04/28.60	2.80/23.81	Topical disinfectant	Bacteriostatic action	[28]
Mefloquine	3.89/30.04	3.25/29.78	Antimalarial agent	Targeting *Plasmodium falciparum* 80S ribosome	[29]
Nebivolol	4.74/32.87	4.17/32.71	Anti-hypertension	β-blocker	[27]
Sertraline	1.92/38.89	1.67/33.32	Antidepressant	Selective serotonin reuptake inhibitor	[16,17]

## Data Availability

Not applicable.

## Supplementary Materials

The following supporting information can be downloaded at: https://www.mdpi.com/article/10.3390/v14010109/s1, Table S1: a list of the BML-2843-0100 drug library; Figure S1: Chemical structures of sertraline, fluvoxamine and paroxetine in Supplementary Section S1; Supplementary Website: The expression profiles of the serotonin transporter encoded by the SLC6A4 gene in various tissues and cell lines, in Supplementary Section S2.
