# Supplementary material for: Antidepressant Sertraline Is a Broad-Spectrum Inhibitor of Enteroviruses Targeting Viral Entry through Neutralization of Endolysosomal Acidification
Source: Viruses. 2022 Jan 8;14(1):109. doi: 10.3390/v14010109 (PMC8780434; doi:10.3390/v14010109)
Supplement: Supplementary file 1 [file viruses-14-00109-s001.zip › Figure S1 and Website.pdf]

Supplementary Materials
Section S1

(**A**) (**B**) (**C**)

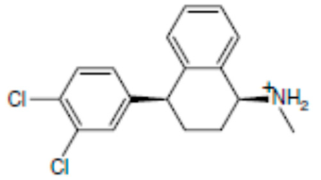

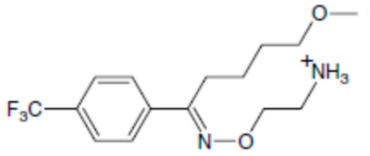

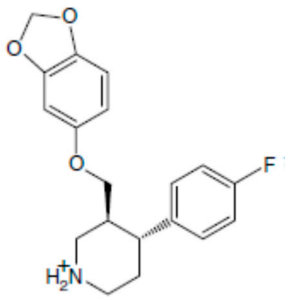

Supplementary Figure S1. Chemical structures of (**A**) sertraline, (**B**) fluvoxamine and (**C**) paroxetine.

Section S2

Supplementary Website. The expression profiles of the serotonin transporter encoded by the SLC6A4 gene in various tissues and cell lines can be referred to the following website A and B, respectively.

A. Tissue expression of SLC6A4 - Summary - The Human Protein Atlas (https://www.proteinatlas.org/ENSG00000108576-SLC6A4/tissue (accessed on 3 January 2022))
B. Cell line - SLC6A4 - The Human Protein Atlas (https://www.proteinatlas.org/ENSG00000108576-SLC6A4/cell+line (accessed on 3 January 2022))

Form the "B" website, it is noted that HeLa cells showed minimal expression of the SLC6A4 gene. While RD cell line is not in the list in the "B' website, the SLC6A4 expression is minimal in muscle tissues revealed in the "A" website.
