# Supplementary material for: Antidepressant Sertraline Is a Broad-Spectrum Inhibitor of Enteroviruses Targeting Viral Entry through Neutralization of Endolysosomal Acidification
Source: Viruses. 2022 Jan 8;14(1):109. doi: 10.3390/v14010109 (PMC8780434; doi:10.3390/v14010109)
Supplement: Supplementary file 1 [file viruses-14-00109-s001.zip › Table S1.pdf]

Table S1. Screen-Well® FDA Approved Drug Library II

BML-2843 Version 1.0

For Research Use Only

#	Plate Location	Cat No.	Name	CAS #	MW	Conc
1	1-A02	BML-A190	Clindamycin·HCl	21462-39-5	461.4	10mM
2	1-A03	BML-DL567	Felbamate	25451-15-4	238.2	10mM
3	1-A04	BML-A195	Cyclosporine A	59865-13-3	1202.6	10mM
4	1-A05	BML-DL568	Donepezil·HCl	120011-70-3	416	10mM
5	1-A06	BML-A240	Lincomycin·HCl	7179-49-9	443	10mM
6	1-A07	BML-A249	Mycophenolic Acid	24280-93-1	320.3	10mM
7	1-A08	BML-A275	Sirolimus (Rapamycin)	53123-88-9	914.2	10mM
8	1-A09	BML-A280	Spectinomycin·HCl Pentahydrate	22189-32-8	495.3	10mM
9	1-A10	BML-AC105	Amiodarone·HCl	19774-82-4	681.8	10mM
10	1-A11	ALX-550-273	Nicardipine·HCl	54527-84-3	516	10mM
11	1-B02	BML-AC109	Pimozide	2062-78-4	461.6	10mM
12	1-B03	ALX-550-253	Loperamide·HCl	34552-83-5	513.5	10mM
13	1-B04	ALX-550-301	Tolbutamide	64-77-7	270.3	10mM
14	1-B05	ALX-550-271	Glipizide	29094-61-9	445.5	10mM
15	1-B06	BML-AC121	Phentolamine·HCl	73-05-2	317.8	10mM
16	1-B07	BML-AC122	Quinine·HCl·H2O	6119-47-7	360.9	10mM
17	1-B08	BML-AC124	Propafenone·HCl	34183-22-7	377.9	10mM
18	1-B09	BML-AC125	Phenytoin	57-41-0	252.3	10mM
19	1-B10	BML-AC127	Procainamide·HCl	614-39-1	271.8	10mM
20	1-B11	BML-AC128	Lidocaine·HCl·H2O	73-78-9	270.8	10mM
21	1-C02	BML-AC130	Flecainide Acetate	54143-56-5	474.4	10mM
22	1-C03	ALX-350-125	Rosiglitazone	122320-73-4	357.4	10mM
23	1-C04	BML-DL570	Amantadine·HCl	665-66-7	187.7	10mM
24	1-C05	BML-AC146	Prazosin·HCl	19237-84-4	419.9	10mM
25	1-C06	ALX-550-087	Clonidine·HCl	4205-91-8	266.6	10mM
26	1-C07	BML-AC153	Guanabenz Acetate	23256-50-0	291.1	10mM
27	1-C08	BML-AC164	Dihydroergotamine Mesylate	6190-39-2	679.8	10mM
28	1-C09	BML-DL564	Emtricitabine	143491-57-0	247.3	10mM
29	1-C10	BML-AC169	Betaxolol·HCl	63659-19-8	343.9	10mM
30	1-C11	ALX-550-322	Caffeine	58-08-2	194.2	10mM
31	1-D02	BML-AC172	(S)-Timolol Maleate	26921-17-5	432.5	10mM
32	1-D03	BML-AC176	Salbutamol Hemisulfate	51022-70-9	576.7	10mM
33	1-D04	BML-AC181	Pindolol	13523-86-9	248.3	10mM
34	1-D05	BML-AC185	Dobutamine·HCl	49745-95-1	337.8	10mM
35	1-D06	BML-AC192	Sotalol·HCl	959-24-0	308.8	10mM
36	1-D07	BML-AR111	Maprotiline·HCl	10347-81-6	313.9	10mM
37	1-D08	ALX-550-092	Pilocarpine·HCl	54-71-7	244.7	10mM
38	1-D09	BML-AC218	Ipratropium·Br	22254-24-6	412.4	10mM
39	1-D10	ALX-550-096	Tropicamide	1508-75-4	284.4	10mM
40	1-D11	BML-AC232	Pancuronium·2Br	15500-66-0	732.7	10mM
41	1-E02	BML-AC238	Ivermectin	70288-86-7	875.1	10mM
42	1-E03	BML-AC250	Haloperidol	52-86-8	375.9	10mM
43	1-E04	BML-AC280	Cimetidine	51481-61-9	252.3	10mM
44	1-E05	BML-DL555	Zonisamide	68291-97-4	212.2	10mM
45	1-E06	ALX-430-153	Zoledronic Acid Monohydrate	165800-06-6	290.1	10mM
46	1-E07	BML-AC322	Naltrexone·HCl	16676-29-2	377.9	10mM
47	1-E08	BML-DL560	Zolmitriptan	139264-17-8	287.4	10mM
48	1-E09	ALX-550-363	Memantine·HCl	41100-52-1	215.8	10mM
49	1-E10	BML-AC501	Riluzole·HCl	1744-22-5	270.7	10mM
50	1-E11	ALX-400-044	Propofol	2078-54-8	178.3	10mM
51	1-F02	BML-AC615	Aminophylline	317-34-0	210.2	10mM
52	1-F03	BML-DL561	Nateglinide	105816-04-4	317.4	10mM
53	1-F04	BML-AC729	(±) Isoproterenol·HCl	51-30-9	247.7	10mM
54	1-F05	ALX-550-135	Acetylcholine Chloride	60-31-1	181.7	10mM
55	1-F06	BML-AC735	Atropine Sulfate Monohydrate	5908-99-6	694.8	10mM
56	1-F07	BML-AC748	Apomorphine·HCl Hemihydrate	41372-20-7	312.8	10mM

#	Plate Location	Cat No.	Name	CAS #	MW	Conc
57	1-F08	ALX-270-171	Chlorpromazine·HCl	69-09-0	355.3	10mM
58	1-F09	BML-AC753	Fluphenazine·HCl	146-56-5	510.4	10mM
59	1-F10	BML-AC758	Risperidone	106266-06-2	410.5	10mM
60	1-F11	BML-AC763	Diphenhydramine·HCl	147-24-0	291.8	10mM
61	1-G02	BML-AC765	Promethazine·HCl	58-33-3	320.9	10mM
62	1-G03	BML-AC766	Ranitidine·HCl	66357-59-3	350.9	10mM
63	1-G04	BML-AC808	Epinephrine (L-(-)-Epinephrine-(+)-Bitartrate)	51-43-4	333.3	10mM
64	1-G05	ALX-550-076	Norepinephrine Bitartrate Monohydrate	108341-18-0	337.3	10mM
65	1-G06	BML-DL557	Quetiapine Fumarate	111974-72-2	883.1	10mM
66	1-G07	BML-AC813	Imipramine·HCl	113-52-0	316.9	10mM
67	1-G08	BML-AC814	Amoxapine	14028-44-5	313.8	10mM
68	1-G09	BML-AC840	Metoclopramide·HCl	7232-21-5	336.3	10mM
69	1-G10	BML-AC893	Nalbuphine·HCl Dihydrate	23277-43-2	429.9	10mM
70	1-G11	BML-AC911	Carbachol (Carbamylcholine) Chloride	51-83-2	182.7	10mM
71	1-H02	BML-AC925	Famotidine	76824-35-6	337.5	10mM
72	1-H03	BML-AC986	Isoniazid	54-85-3	137.1	10mM
73	1-H04	BML-AC993	Ticlopidine·HCl	53885-35-1	300.2	10mM
74	1-H05	BML-DL247	Clemastine Fumarate	14976-57-9	460	10mM
75	1-H06	BML-DL248	Vardenafil	224785-90-4	488.6	10mM
76	1-H07	BML-DL249	Linezolid	165800-03-3	337.4	10mM
77	1-H08	BML-DL250	Docetaxel (Taxotere)	114977-28-5	807.9	10mM
78	1-H09	BML-DL251	Olopatadine	113806-05-6	337.4	10mM
79	1-H10	BML-DL252	Tolcapone	134308-13-7	273.2	10mM
80	1-H11	BML-DL254	Olmesartan	144689-24-7	446.5	10mM
81	2-A02	BML-DL255	Nisoldipine	63675-72-9	388.4	10mM
82	2-A03	BML-DL257	Olanzapine	132539-06-1	312.4	10mM
83	2-A04	BML-G226	Lovastatin	75330-75-5	404.5	10mM
84	2-A05	ALX-430-160	Lamotrigine	84057-84-1	256.1	10mM
85	2-A06	BML-DL572	Azathioprine	446-86-6	277.3	10mM
86	2-A07	BML-DL261	Sildenafil Citrate	171599-83-0	666.7	10mM
87	2-A08	BML-DL262	Atovaquone	95233-18-4	366.8	10mM
88	2-A09	BML-DL208	Sertaconazole	99592-32-2	437.8	10mM
89	2-A10	BML-DL191	Cefepime·HCl Hydrate	123171-59-5	571.5	10mM
90	2-A11	BML-DL178	Aripiprazole	129722-12-9	448.4	10mM
91	2-B02	BML-DL264	Candesartan	139481-59-7	440.5	10mM
92	2-B03	BML-DL101	Butenafine·HCl	101827-46-7	353.9	10mM
93	2-B04	BML-DL200	Dorzolamide·HCl	130693-82-2	360.9	10mM
94	2-B05	BML-DL212	Escitalopram	128196-01-0		10mM
95	2-B06	BML-DL209	Eprosartan Mesylate	144143-96-4	520.6	10mM
96	2-B07	BML-DL217	Entacapone	130929-57-6	305.3	10mM
97	2-B08	BML-AP302	Bleomycin Sulfate	9041-93-4	1512.6	10mM
98	2-B09	BML-AR102	Guanfacine·HCl	29110-48-3	282.6	10mM
99	2-B10	BML-AR103	Tizanidine·HCl	64461-82-1	290.2	10mM
100	2-B11	BML-AR112	Carvedilol	72956-09-3	406.5	10mM
101	2-C02	BML-B100	Flumazenil	78755-81-4	303.3	10mM
102	2-C03	BML-DL269	Gefitinib	184475-35-2	446.9	10mM
103	2-C04	ALX-270-492	Imatinib Mesylate	220127-57-1	589.7	10mM
104	2-C05	ALX-380-260	Idarubicin·HCl	57852-57-0	534	10mM
105	2-C06	BML-DL273	Montelukast·Na	151767-02-1	608.2	10mM
106	2-C07	BML-DL573	Exemestane	107868-30-4	296.4	10mM
107	2-C08	BML-PG007	Dinoprostone	363-24-6	352.5	10mM
108	2-C09	ALX-270-432	Metformin·HCl	1115-70-4	165.6	10mM
109	2-C10	BML-DL277	Anagrelide	68475-42-3	256.1	10mM
110	2-C11	BML-DL278	Dofetilide	115256-11-6	441.6	10mM
111	2-D02	BML-DL279	Erlotinib	183321-74-6	393.4	10mM
112	2-D03	BML-C110	Tacrine·HCl	1684-40-8	234.7	10mM
113	2-D04	ALX-550-336	Galantamine·HBr	1953-04-4	368.3	10mM
114	2-D05	BML-CA200	Amiloride·HCl·2H2O	17440-83-4	302.1	10mM
115	2-D06	BML-CA202	Amlodipine	88150-42-9	408.9	10mM

#	Plate Location	Cat No.	Name	CAS #	MW	Conc
116	2-D07	ALX-550-214	Diltiazem·HCl	33286-22-5	451	10mM
117	2-D08	ALX-550-091	Nifedipine	21829-25-4	346.3	10mM
118	2-D09	ALX-550-277	Nimodipine	66085-59-4	418.5	10mM
119	2-D10	ALX-550-306	Verapamil·HCl	152-11-4	491.1	10mM
120	2-D11	BML-CA234	Gabapentin	60142-96-3	171.2	10mM
121	2-E02	BML-CA236	Felodipine	72509-76-3	384.3	10mM
122	2-E03	ALX-430-039	Phenoxybenzamine·HCl	63-92-3	340.3	10mM
123	2-E04	ALX-550-310	Trifluoperazine·HCl	440-17-5	480.4	10mM
124	2-E05	BML-DL281	Latanoprost	130209-82-4	432.6	10mM
125	2-E06	BML-DL574	Alfuzosin	81403-80-7	389.5	10mM
126	2-E07	BML-D102	Bromocriptine Mesylate	22260-51-1	750.7	10mM
127	2-E08	BML-D107	Clozapine	5786-21-0	326.8	10mM
128	2-E09	BML-DL575	Acitretin	55079-83-9	326.4	10mM
129	2-E10	BML-DM200	Calcitriol	32222-06-3	416.6	10mM
130	2-E11	BML-EI107	Ketoconazole	65277-42-1	531.4	10mM
131	2-F02	BML-EI121	Cromolyn·Na (Disodium Cromoglycate)	15826-37-6	512.3	10mM
132	2-F03	BML-EI125	Capsaicin	404-86-4	305.5	10mM
133	2-F04	BML-EI126	Dexamethasone	50-02-2	392.5	10mM
134	2-F05	BML-EI127	Dipyridamole	58-32-2	504.6	10mM
135	2-F06	BML-EI128	Ethacrynic Acid	58-54-8	303.1	10mM
136	2-F07	ALX-270-086	Indomethacin	53-86-1	357.8	10mM
137	2-F08	ALX-270-102	Naproxen	22204-53-1	230.3	10mM
138	2-F09	BML-EI164	Ibuprofen	15687-27-1	206.3	10mM
139	2-F10	BML-EI168	Bumetanide	28395-03-1	364.4	10mM
140	2-F11	ALX-380-035	Neomycin Sulfate	1405-10-3	908.9	10mM
141	2-G02	BML-EI206	Auranofin	34031-32-8	678.5	10mM
142	2-G03	ALX-270-212	Captopril	62571-86-2	217.3	10mM
143	2-G04	BML-EI217	Tranylcypromine Hemisulfate	13492-01-8	182.2	10mM
144	2-G05	ALX-430-040	Piroxicam	36322-90-4	331.3	10mM
145	2-G06	BML-DL577	Moxifloxacin·HCl	186826-86-8	437.9	10mM
146	2-G07	BML-EI265	Carbidopa	28860-95-9	226.2	10mM
147	2-G08	BML-EI288	Ketoprofen	22071-15-4	254.3	10mM
148	2-G09	BML-EI292	Meloxicam	71125-38-7	351.4	10mM
149	2-G10	BML-EI318	Terbinafine·HCl	78628-80-5	327.9	10mM
150	2-G11	BML-EI320	Sodium Phenylbutyrate	1716-12-7	186.2	10mM
151	2-H02	BML-G244	Simvastatin	79902-63-9	418.6	10mM
152	2-H03	BML-DL282	Goserelin Acetate	145781-92-6	1269.4	10mM
153	2-H04	BML-GR243	Raloxifene·HCl	82640-04-8	510.1	10mM
154	2-H05	ALX-380-071	Rifampin (Rifampicin)	13292-46-1	822.9	10mM
155	2-H06	BML-GR307	Etoposide	33419-42-0	588.6	10mM
156	2-H07	BML-GR311	Mitomycin C	50-07-7	334.3	10mM
157	2-H08	BML-GR314	Delavirdine Mesylate	147221-93-0	522.7	10mM
158	2-H09	ALX-380-043	Daunorubicin·HCl	23541-50-6	564	10mM
159	2-H10	BML-GR319	Doxorubicin·HCl	25316-40-9	580	10mM
160	2-H11	BML-DL578	Cetirizine 2HCl	83881-52-1	461.8	10mM
161	3-A02	BML-DL284	Lapatinib Ditosylate	388082-77-7	925.5	10mM
162	3-A03	BML-GR240	Pioglitazone·HCl	112529-15-4	392.9	10mM
163	3-A04	BML-DL288	Rivastigmine Tartrate	129101-54-8	400.4	10mM
164	3-A05	BML-DL236	Ergotamine Tartrate	379-79-3	656.7	10mM
165	3-A06	ALX-430-105	Sulindac	38194-50-2	356.4	10mM
166	3-A07	ALX-550-304	Valproic Acid	99-66-1	144.2	10mM
167	3-A08	BML-DL547	Calcipotriene	112965-21-6	412.6	10mM
168	3-A09	BML-DL294	Zafirlukast	107753-78-6	575.7	10mM
169	3-A10	BML-DL295	Zileuton	111406-87-2	236.3	10mM
170	3-A11	BML-DL297	Bortezomib	179324-69-7	384.2	10mM
171	3-B02	ALX-550-260	Diazoxide	364-98-7	230.7	10mM
172	3-B03	BML-KC120	Glyburide	10238-21-8	494	10mM
173	3-B04	BML-KC125	Minoxidil	38304-91-5	209.3	10mM
174	3-B05	ALX-550-216	Tolazamide	1156-19-0	311.4	10mM

#	Plate Location	Cat No.	Name	CAS #	MW	Conc
175	3-B06	BML-DL298	Bexarotene	153559-49-0	348.5	10mM
176	3-B07	BML-DL579	Tranexamic Acid	1197-18-8	157.2	10mM
177	3-B08	BML-DL299	Celecoxib	169590-42-5	381.4	10mM
178	3-B09	BML-DL172	Levetiracetam	102767-28-2	170.2	10mM
179	3-B10	BML-DL300	Letrozole	112809-51-5	285.3	10mM
180	3-B11	BML-DL144	Anastrozole	120511-73-1	293.4	10mM
181	3-C02	ALX-270-476	Bicalutamide	90357-06-5	430.4	10mM
182	3-C03	BML-DL102	Clindamycin Palmitate·HCl	25507-04-4	699.9	10mM
183	3-C04	ALX-270-288	Vorinostat	149647-78-9	264.3	10mM
184	3-C05	BML-DL302	Didanosine	69655-05-6	236.2	10mM
185	3-C06	BML-DL199	Dolasetron	115956-12-2	324.4	10mM
186	3-C07	BML-DL192	Enalaprilat Maleate	76095-16-4	492.5	10mM
187	3-C08	ALX-270-466	Fluvastatin·Na	93957-55-2	433.5	10mM
188	3-C09	BML-DL155	Fosinopril·Na	88889-14-9	585.6	10mM
189	3-C10	ALX-480-101	Gemcitabine·HCl	122111-03-9	299.7	10mM
190	3-C11	BML-DL166	Granisetron·HCl	107007-99-8	348.9	10mM
191	3-D02	ALX-400-042	Oxaliplatin	61825-94-3	397.3	10mM
192	3-D03	BML-DL546	Atazanavir	198904-31-3	704.9	10mM
193	3-D04	BML-IM101	Mycophenolate Mofetil	128794-94-5	433.5	10mM
194	3-D05	BML-DL103	Clofarabine	123318-82-1	303.7	10mM
195	3-D06	BML-DL219	Cabergoline	81409-90-7	451.6	10mM
196	3-D07	BML-DL213	Ibandronate·Na Monohydrate	138926-19-9	359.2	10mM
197	3-D08	BML-DL202	Imipenem	64221-86-9	299.4	10mM
198	3-D09	ALX-400-038	Lomustine	13010-47-4	233.7	10mM
199	3-D10	BML-GR111	Adapalene	106685-40-9	412.5	10mM
200	3-D11	BML-DL167	Meropenem	96036-03-2	383.5	10mM
201	3-E02	BML-DL194	Oseltamivir Phosphate	204255-11-8	410.4	10mM
202	3-E03	BML-DL196	Pamidronate Disodium Pentahydrate (Pamidronic	109552-15-0	369.1	10mM
203	3-E04	BML-DL197	Pramipexole Dihydrochloride Monohydrate	191217-81-9	302.3	10mM
204	3-E05	BML-DL310	Triptorelin Acetate	140194-24-7	1311.5	10mM
205	3-E06	BML-DL198	Risredonic Acid	105462-24-6	283.1	10mM
206	3-E07	BML-DL187	Rocuronium Bromide	119302-91-9	609.7	10mM
207	3-E08	BML-NP461	Vinorelbine	71486-22-1	778.9	10mM
208	3-E09	BML-DL210	Salmeterol	893365-50-4	415.6	10mM
209	3-E10	BML-T117	Vincristine Sulfate	2068-78-2	923	10mM
210	3-E11	ALX-430-115	Aspirin (Acetylsalicylic Acid)	50-78-2	180.2	10mM
211	3-F02	BML-DL316	Acyclovir (Acycloguanosine) Zovirax	59277-89-3	225.2	10mM
212	3-F03	BML-DL317	Zidovudine (3'-Azido-3'-Deoxythymidine)	30516-87-1	267.2	10mM
213	3-F04	BML-DL318	Allopurinol	315-30-0	136.1	10mM
214	3-F05	BML-DL320	Altretamine	645-05-6	210.3	10mM
215	3-F06	BML-PR123	**Alendronate·Na Trihydrate**	121268-17-5	325.1	10mM
216	3-F07	BML-DL321	Albendazole	54965-21-8	265.3	10mM
217	3-F08	BML-DL565	Sumatriptan Succinate	103628-48-4	372.4	10mM
218	3-F09	BML-GR341	Amifostine	20537-88-6	214.2	10mM
219	3-F10	BML-DL325	4-Aminosalicylic Acid	65-49-6	153.1	10mM
220	3-F11	ALX-430-110	Mesalamine (5-Aminosalicylic Acid)	89-57-6	153.1	10mM
221	3-G02	BML-DL327	Ampicillin Trihydrate	7177-48-2	403.5	10mM
222	3-G03	BML-AC166	(±)-Atenolol	29122-68-7	266.3	10mM
223	3-G04	BML-DL332	Atracurium Besylate	64228-81-5	1243.5	10mM
224	3-G05	ALX-350-257	Vinblastine Sulfate	143-67-9	909.1	10mM
225	3-G06	BML-DL335	Azithromycin	83905-01-5	749	10mM
226	3-G07	BML-DL336	Aztreonam	78110-38-0	435.4	10mM
227	3-G08	BML-DL337	Betamethasone	378-44-9	392.5	10mM
228	3-G09	BML-DL339	Bisacodyl	603-50-9	361.4	10mM
229	3-G10	BML-DL341	Buspirone·HCl	33386-08-2	422	10mM
230	3-G11	ALX-400-041	Carboplatin	41575-94-4	371.3	10mM
231	3-H02	ALX-550-084	Carbamazepine	298-46-4	236.3	10mM
232	3-H03	BML-DL347	Cefotaxime Acid	63527-52-6	455.5	10mM
233	3-H04	BML-DL348	Ceftazidime	72558-82-8	346.6	10mM

#	Plate Location	Cat No.	Name	CAS #	MW	Conc
234	3-H05	BML-DL349	Chloramphenicol	56-75-7	323.1	10mM
235	3-H06	ALX-400-049	Chlorambucil	305-03-3	304.2	10mM
236	3-H07	BML-DL352	Chlorpheniramine Maleate	113-92-8	390.9	10mM
237	3-H08	BML-DL353	Chloroquine Diphosphate	50-63-5	515.9	10mM
238	3-H09	BML-T115	Thalidomide	50-35-1	258.2	10mM
239	3-H10	ALX-380-287	Ciprofloxacin	85721-33-1	331.3	10mM
240	3-H11	BML-NS112	Citalopram·HBr	59729-32-7	405.3	10mM
241	4-A02	BML-DL357	Clarithromycin	81103-11-9	748	10mM
242	4-A03	BML-DL360	Clomiphene Citrate	50-41-9	598.1	10mM
243	4-A04	BML-AC1290	Clopidogrel Hydrogen Sulfate	135046-48-9	419.9	10mM
244	4-A05	BML-DL363	Clobetasol Propionate	25122-46-7	467	10mM
245	4-A06	BML-AC1386	Orphenadrine Citrate	4682-36-4	461.5	10mM
246	4-A07	BML-DL366	Crotamiton	483-63-6	203.3	10mM
247	4-A08	ALX-400-051	Cyclophosphamide (Free Base)	6055-19-2	279.1	10mM
248	4-A09	BML-DL370	Cytarabine	147-94-4	243.2	10mM
249	4-A10	BML-DL371	Dacarbazine	4342-03 4	182.2	10mM
250	4-A11	BML-DL372	Danazol	17230-88-5	337.5	10mM
251	4-B02	BML-DL154	Desloratadine	100643-71-8	310.8	10mM
252	4-B03	BML-DL374	Dextromethorphan	125-71-3	271.4	10mM
253	4-B04	BML-DL375	Diclofenac·Na Salt	15307-79-6	318.3	10mM
254	4-B05	BML-DL376	Zalcitabine (2',3'-Dideoxycytidine)	7481-89-2	211.2	10mM
255	4-B06	BML-DL378	Diflunisal	22494-42-4	250.2	10mM
256	4-B07	BML-DL379	Disulfiram	97-77-8	296.5	10mM
257	4-B08	BML-DL204	Doxazosin Mesylate	77883-43-3	547.6	10mM
258	4-B09	BML-DL381	Doxycycline Monohydrate	17086-28-1	480.9<br>462.45	10mM
259	4-B10	BML-PI152	Enalapril	75847-73-3	376.5	10mM
260	4-B11	BML-DL386	Esomeprazole Potassium	161796-84-5	383.5	10mM
261	4-C02	BML-BL093	Estradiol	50-28-2	272.4	10mM
262	4-C03	BML-BL090	Estrone	53-16-7	270.4	10mM
263	4-C04	BML-DL390	Etidronate Disodium	7414-83-7	250	10mM
264	4-C05	BML-DL392	Famciclovir	104227-87-4	321.3	10mM
265	4-C06	BML-DL395	Fenoldopam Mesylate	67227-57-0	401.9	10mM
266	4-C07	BML-DL396	Fenoprofen Calcium	53746-45-5	558.6	10mM
267	4-C08	ALX-270-481	Fenofibrate	49562-28-9	360.8	10mM
268	4-C09	ALX-270-491	Finasteride	98319-26-7	372.5	10mM
269	4-C10	ALX-480-099	Fluorouracil (5-Fluorouracil)	51-21-8	130.1	10mM
270	4-C11	BML-DL400	Flurbiprofen	5104-49-4	244.3	10mM
271	4-D02	BML-DL581	Amitriptyline·HCl	549-18-8	313.9	10mM
272	4-D03	BML-DL403	Floxuridine	50-91-9	246.2	10mM
273	4-D04	BML-DL404	Fluocinolone Acetonide	67-73-2	452.5	10mM
274	4-D05	BML-DL406	Flutamide	13311-84-7	276.2	10mM
275	4-D06	BML-DL407	Fluconazole	86386-73-4	306.3	10mM
276	4-D07	BML-DL410	Furosemide	54-31-9	330.7	10mM
277	4-D08	BML-DL411	Ganciclovir	82410-32-0	255.2	10mM
278	4-D09	ALX-380-291	Gatifloxacin	112811-59-3	375.4	10mM
279	4-D10	BML-DL413	Gentamycin Sulfate	1405-41-0	570	10mM
280	4-D11	BML-DL414	Gemfibrozil	25812-30-0	250.3	10mM
281	4-E02	BML-DL416	Glimepiride	93479-97-1	490.6	10mM
282	4-E03	BML-DL419	Hydrocortisone	50-23-7	362.5	10mM
283	4-E04	BML-DL420	Hydrocortisone Acetate	50-03-3	404.5	10mM
284	4-E05	BML-DL422	Idoxuridine	54-42-2	354.1	10mM
285	4-E06	BML-DL423	Ifosfamide	3778-73-2	261.1	10mM
286	4-E07	ALX-420-039	Imiquimod	99011-02-6	240.3	10mM
287	4-E08	BML-DL425	Indapamide	26807-65-8	365.8	10mM
288	4-E09	BML-DL427	Itraconazole	84625-61-6	705.6	10mM
289	4-E10	BML-DL430	Levonorgestrel	797-63-7	312.4	10mM
290	4-E11	ALX-380-292	Levofloxacin·HCl	177325-13-2	415.8	10mM
291	4-F02	ALX-430-095	Leflunomide	75706-12-6	270.2	10mM
292	4-F03	BML-DL434	Lisinopril·2H2O	83915-83-7	441.5	10mM

#	Plate Location	Cat No.	Name	CAS #	MW	Conc
293	4-F04	BML-DL436	Loratadine	79794-75-5	382.9	10mM
294	4-F05	BML-DL438	Losartan Potassium	124750-99-8	461	10mM
295	4-F06	BML-DL439	Mebendazole	31431-39-7	295.3	10mM
296	4-F07	BML-DL440	Medroxyprogesterone Acetate	71-58-9	386.5	10mM
297	4-F08	BML-DL441	Mefenamic Acid	61-68-7	241.3	10mM
298	4-F09	BML-DL442	Melphalan	148-82-3	305.2	10mM
299	4-F10	ALX-550-528	Methyldopa Sesquihydrate (L- A -Methyl-Dopa	41372-08-1	238.2	10mM
300	4-F11	BML-DL444	Methylprednisolone	83-43-2	374.5	10mM
301	4-G02	BML-DL445	Metoprolol Tartrate	56392-17-7	684.8	10mM
302	4-G03	BML-DL446	Methimazole	60-56-0	114.2	10mM
303	4-G04	BML-DL447	Metronidazole	443-48-1	171.2	10mM
304	4-G05	BML-DL449	Minocycline	10118-90-8	457.48	10mM
305	4-G06	ALX-400-050	Mitoxantrone·HCl	70476-82-3	517.4	10mM
306	4-G07	BML-T104	Paclitaxel (Taxol)	33069-62-4	853.9	10mM
307	4-G08	BML-DL454	Nabumetone	42924-53-8	228.3	10mM
308	4-G09	BML-DL455	Naphazoline·HCl	550-99-2	246.7	10mM
309	4-G10	BML-DL456	Nefazodone·HCl	82752-99-6	506.5	10mM
310	4-G11	BML-DL458	Norethindrone	68-22-4	298.4	10mM
311	4-H02	ALX-380-295	Norfloxacin	70458-96-7	319.3	10mM
312	4-H03	BML-DL460	Nystatin	1400-61-9	926.1	10mM
313	4-H04	ALX-380-297	Ofloxacin	82419-36-1	361.4	10mM
314	4-H05	BML-DL462	Omeprazole	73590-58-6	345.4	10mM
315	4-H06	BML-DL463	Oxcarbazepine	28721-07-5	252.3	10mM
316	4-H07	BML-DL464	Oxiconazole Nitrate	64211-46-7	492.2	10mM
317	4-H08	BML-DL465	Oxacillin·Na	7240-38-2	441.4	10mM
318	4-H09	BML-DL468	Pantoprazole	102625-70-7	383.4	10mM
319	4-H10	BML-NS710	Paroxetine·HCl	78246-49-8	365.8	10mM
320	4-H11	BML-DL472	Penciclovir	39809-25-1	253.3	10mM
321	5-A02	ALX-270-112	Pentoxifylline	6493-05 6	278.3	10mM
322	5-A03	BML-DL474	Penicillin V Potassium	132-98-9	388.5	10mM
323	5-A04	BML-DL476	Piperacillin	61477-96-1	517.6	10mM
324	5-A05	BML-DL479	Prednisolone	50-24-8	360.4	10mM
325	5-A06	BML-DL480	Progesterone	57-83-0	314.5	10mM
326	5-A07	BML-DL481	Procarbazine·HCl	366-70-1	257.8	10mM
327	5-A08	BML-DL483	Prednisone	53-03-2	358.4	10mM
328	5-A09	BML-DL485	Primaquine Phosphate	63-45-6	455.3	10mM
329	5-A10	BML-DL486	Praziquantel	55268-74-1	312.4	10mM
330	5-A11	BML-DL489	Quinapril·HCl	82586-55-8	475	10mM
331	5-B02	BML-DL490	Ranolazine·2HCl	95635-56-6	500.5	10mM
332	5-B03	BML-DL491	Ramipril	87333-19-5	416.5	10mM
333	5-B04	BML-DL492	Ribavirin	36791-04-5	441.4	10mM
334	5-B05	BML-DL566	Nelfinavir Mesylate	159989-65-8	663.9	10mM
335	5-B06	BML-DL494	Rimantadine·HCl	1501-84-4	179.3	10mM
336	5-B07	BML-ST405	Propranolol·HCl	318-98-9	295.8	10mM
337	5-B08	ALX-550-094	Scopolamine·HBr	114-49-8	438.3	10mM
338	5-B09	BML-DL504	Spironolactone	52-01-7	416.6	10mM
339	5-B10	ALX-380-277	Streptomycin Sulfate	3810-74-0	1457.4	10mM
340	5-B11	BML-DL508	Sulfadiazine	68-35-9	250.3	10mM
341	5-C02	BML-DL510	Sulfasalazine	599-79-1	398.4	10mM
342	5-C03	BML-DL160	Tamsulosin·HCl	106463-17-6	445	10mM
343	5-C04	BML-DL511	Telmisartan	144701-48-4	514.6	10mM
344	5-C05	BML-DL513	Terazosin·HCl	63590-64-7	423.9	10mM
345	5-C06	BML-DL514	Tetracycline	60-54-8	444.4	10mM
346	5-C07	ALX-420-044	Temozolomide	85622-93-1	194.2	10mM
347	5-C08	BML-DL516	Tinidazole	19387-91-8	247.3	10mM
348	5-C09	ALX-380-018	Tobramycin	32986-56-4	467.5	10mM
349	5-C10	ALX-350-133	Topotecan·HCl	119413-54-6	457.9	10mM
350	5-C11	BML-DL519	Toremifene Base	89778-26-7	406	10mM
351	5-D02	BML-DL521	Tolmetin·Na	64490-92-2	315.3	10mM

#	Plate Location	Cat No.	Name	CAS #	MW	Conc
352	5-D03	BML-DL582	Amoxicillin	26787-78-0	365.4	10mM
353	5-D04	BML-DL523	Tramadol·HCl	22204-88-2	299.8	10mM
354	5-D05	BML-DL525	Trimethoprim	738-70-5	290.3	10mM
355	5-D06	BML-DL527	Valacyclovir·HCl	124832-27-5	360.8	10mM
356	5-D07	BML-DL528	Vecuronium Bromide	50700-72-6	637.7	10mM
357	5-D08	BML-NS114	Venlafaxine·HCl	99300-78-4	313.9	10mM
358	5-D09	BML-NA139	Bupivacaine·HCl	14252-80-3	324.9	10mM
359	5-D10	BML-NH106	Ketotifen Fumarate	34580-14-8	425.5	10mM
360	5-D11	ALX-550-055	Naloxone·HCl	357-08-4	363.9	10mM
361	5-E02	BML-NS140	Fluoxetine·HCl	56296-78-7	345.8	10mM
362	5-E03	BML-NS145	Ondansetron	116002-70-1	293.4	10mM
363	5-E04	BML-DL562	Tiotropium Bromide Monohydrate	136310-93-5	472.4	10mM
364	5-E05	BML-NS835	Thioridazine·HCl	130-61-0	407	10mM
365	5-E06	BML-DL583	Amrinone	60719-84-8	187.2	10mM
366	5-E07	ALX-270-083	Milrinone	78415-72-2	211.2	10mM
367	5-E08	BML-PG006	Alprostadil	745-65-3	354.5	10mM
368	5-E09	BML-PG051	Misoprostol	59122-46-2	382.5	10mM
369	5-E10	BML-PI146	Argatroban	74863-84-6	508.6	10mM
370	5-E11	BML-PI153	Cilastatin·Na	81129-83-1	380.4	10mM
371	5-F02	BML-DL584	Butoconazole Nitrate	64872-77-1	474.8	10mM
372	5-F03	BML-S510	Mifepristone	84371-65-3	429.6	10mM
373	5-F04	BML-S515	Megestrol Acetate	595-33-5	384.5	10mM
374	5-F05	ALX-550-095	Tamoxifen Citrate	54965-24-1	563.6	10mM
375	5-F06	BML-DL533	Aprepitant	170729-80-3	534.4	10mM
376	5-F07	BML-DL534	Bosentan	147536-97-8	551.7	10mM
377	5-F08	BML-DL535	Efavirenz	154598-52-4	315.7	10mM
378	5-F09	BML-SL230	Miglustat (N-Butyldeoxynojirimycin·HCl)	210110-90-0	255.7	10mM
379	5-F10	BML-DL104	Fulvestrant	129453-61-8	606.8	10mM
380	5-F11	BML-DL107	Esmolol	103598-03-4	295.4	10mM
381	5-G02	BML-DL585	Capecitabine	154361-50-9	359.4	10mM
382	5-G03	BML-DL234	Succinylcholine Chloride·2H2O	6101-15-1	397.3	10mM
383	5-G04	BML-DL136	Cyproheptadine·HCl Sesquihydrate	41354-29-4	350.9	10mM
384	6-A02	BML-AC2300	Abacavir Sulfate	188062-50-2	286.3	10mM
385	6-A03	BML-AC3000	Acamprosate	77337-73-6	181.2	10mM
386	6-A04	BML-AC2310	Acarbose	56180-94-0	645.6	10mM
387	6-A05	BML-AC2003	Acebutolol·HCl	34381-68-5	372.9	10mM
388	6-A06	BML-AC2337	Acetaminophen	103-90-2	151.2	10mM
389	6-A07	BML-AC3001	Acetazolamide	59-66-5	222.3	10mM
390	6-A08	BML-AC3002	Acetohexamide	968-81-0	324.4	10mM
391	6-A09	BML-AC3003	Acetohydroxamic Acid	546-88-3	75.1	10mM
392	6-A10	BML-AC3004	Acetylcysteine	616-91-1	163.2	10mM
393	6-A11	BML-AC3005	Acrivastine	87848-99-5	348.4	2mM
394	6-B02	BML-AC3006	Adefovir Dipivoxil	142340-99-6	501.5	10mM
395	6-B03	BML-AC589	Adenosine	58-61-7	267.2	10mM
396	6-B04		Blank			
397	6-B05	BML-AC3008	Alitretinoin	5300-03-8	300.4	10mM
398	6-B06	BML-AC3009	Almotriptan	181183-52-8	335.5	10mM
399	6-B07	BML-AC3010	Alosetron·HCl	122852-69-1	294.4	10mM
400	6-B08		Blank			
401	6-B09	BML-AC3012	Ambrisentan	177036-94-1	378.4	10mM
402	6-B10	BML-AC3013	Amcinonide	51022-69-6	502.6	10mM
403	6-B11	ALX-380-266	Amikacin Disulfate	39831-55-5	781.8	10mM
404	6-C02	BML-AC3014	Aminocaproic Acid	60-32-2	131.2	10mM
405	6-C03	BML-AC3015	Aminohippurate·Na	94-16-6	216.2	10mM
406	6-C04	BML-AC3016	Aminolevulinic Acid·HCl	5451-09-2	167.6	10mM
407	6-C05	BML-AC3017	Amlexanox	68302-57-8	298.3	10mM
408	6-C06	ALX-380-280	Amphotericin B	1397-89-3	924.1	10mM
409	6-C07	BML-AC2011	Arsenic Trioxide	1327-53-3	197.8	10mM
410	6-C08	BML-AC3019	Artemether	71963-77-4	298.4	10mM

#	Plate Location	Cat No.	Name	CAS #	MW	Conc
411	6-C09	BML-AC3020	Articaine·HCl	23964-57-0	320.8	10mM
412	6-C10	ALX-460-001	L-Ascorbic Acid	50-81-7	176	10mM
413	6-C11	BML-AC3021	Asenapine Maleate	85650-56-2	401.8	10mM
414	6-D02	BML-AC3022	Atomoxetine·HCl	82248-59-7	291.8	10mM
415	6-D03	BML-AC3023	Atorvastatin Calcium	134523-03-8	577.7	10mM
416	6-D04	BML-AC3024	Azacitidine	320-67-2	244.2	10mM
417	6-D05	BML-AC3025	Azelaic Acid	123-99-9	188.2	10mM
418	6-D06	BML-AC3026	Azelastine·HCl	79307-93-0	418.4	10mM
419	6-D07	BML-AC3027	Bacitracin	1405-87-4	1422.7	10mM
420	6-D08	BML-EA128	Baclofen	1134-47-0	213.7	10mM
421	6-D09	BML-AC3028	Balsalazide	80573-04-2	354.3	10mM
422	6-D10	BML-AC3029	Beclomethasone Dipropionate	5534-09-8	521	10mM
423	6-D11	BML-AC3030	Benazepril·HCl	86541-74-4	461	10mM
424	6-E02	BML-AC1523	Bendamustine·HCl	3543-75-7	394.7	10mM
425	6-E03	BML-AC3031	Bendroflumethiazide	73-48-3	421.4	10mM
426	6-E04	ALX-550-143	Benztropine Mesylate	132-17-2	403.5	10mM
427	6-E05	BML-AC3032	Betaine	107-43-7	117.2	10mM
428	6-E06	BML-AC3033	Bethanechol Chloride	590-63-6	196.7	10mM
429	6-E07	BML-RA121	Bimatoprost	155206-00-1	415.6	10mM
430	6-E08	BML-AC3034	Biperiden·HCl	1235-82-1	347.9	10mM
431	6-E09	BML-AC3035	Bisoprolol Fumarate	104344-23-2	767	10mM
432	6-E10	BML-AC3036	Brimonidine	59803-98-4	292.1	10mM
433	6-E11	BML-AC3037	Bromfenac	91714-94-2	334.2	10mM
434	6-F02	BML-AC3038	Brompheniramine Maleate	980-71-2	435.3	10mM
435	6-F03	BML-AC3039	Budesonide	51333-22-3	430.5	10mM
436	6-F04	BML-AC2606	Bupropion	34911-55-2	239.7	10mM
437	6-F05	ALX-400-048	Busulfan	55-98-1	246.3	10mM
438	6-F06	BML-AC3040	Butorphanol-(+)-Tartrate (Schedule Iv)	58786-99-5	477.6	10mM
439	6-F07	BML-NP042	Capreomycin Sulfate	1405-36-3	750.8	10mM
440	6-F08	BML-AC3041	Carbinoxamine Maleate	3505-38-2	406.9	10mM
441	6-F09	BML-AC3042	Carglumic Acid	1188-38-1	190.2	10mM
442	6-F10		Blank			
443	6-F11	BML-AC2600	Carmustine	154-93-8	214.1	10mM
444	6-G02	ALX-380-016	Cefaclor	53994-73-3	367.8	10mM
445	6-G03	BML-AC3044	Cefadroxil	66592-87-8	363.4	10mM
446	6-G04	ALX-380-444	Cefazolin·Na	27164-46-1	476.5	10mM
447	6-G05	BML-AC3045	Cefdinir	91832-40-5	395.4	10mM
448	6-G06	BML-AC3046	Cefditoren Pivoxil	117467-28-4	620.7	10mM
449	6-G07	BML-AC3047	Cefixime	79350-37-1	453.5	10mM
450	6-G08	BML-AC3048	Cefotetan Disodium	74356-00-6	619.6	10mM
451	6-G09	BML-AC3049	Cefoxitin·Na	33564-30-6	449.4	10mM
452	6-G10	BML-AC3050	Cefpodoxime Proxetil	87239-81-4	557.6	10mM
453	6-G11	BML-AC3051	Cefprozil	92665-29-7	389.4	10mM
454	6-H02	BML-AC3052	Ceftibuten	97519-39-6	410.4	10mM
455	6-H03	BML-AC3053	Ceftizoxim·Na	68401-82-1	405.4	10mM
456	6-H04	BML-AC3054	Ceftriaxone·Na	104376-79-6	598.6	10mM
457	6-H05	BML-AC3055	Cefuroxime Axetil	64544-07-6	510.5	10mM
458	6-H06	BML-AC3056	Cefuroxime·Na	56238-63-2	446.4	10mM
459	6-H07	BML-AC3057	Cephalexin Monohydrate	15686-71-2	365.4	10mM
460	6-H08	BML-BL053	Chenodiol (Chenodeoxycholic Acid)	474-25-9	392.6	10mM
461	6-H09		Blank			
462	6-H10	BML-AC3059	Chlorhexidine Dihydrochloride	3697-42-5	578.4	10mM
463	6-H11	ALX-500-086	Chlorothiazide	58-94-6	295.7	10mM
464	7-A02	BML-AC3060	Chlorpropamide	94-20-2	276.7	10mM
465	7-A03	BML-AC3061	Chlorthalidone	77-36-1	338.8	10mM
466	7-A04	BML-AC2325	Chlorzoxazone	95-25-0	169.6	10mM
467	7-A05	BML-AC3062	Ciclesonide	126544-47-6	540.7	10mM
468	7-A06	BML-AC3063	Ciclopirox	29342-05-0	207.3	10mM
469	7-A07	BML-AC3064	Cidofovir	113852-37-2	279.2	10mM

#	Plate Location	Cat No.	Name	CAS #	MW	Conc
470	7-A08	BML-PD127	Cilostazol	73963-72-1	369.5	10mM
471	7-A09	BML-AC3065	Cinacalcet·HCl	364782-34-3	393.9	10mM
472	7-A10	BML-AC3066	Cisatracurium Besylate	96946-42-8	1243.8	10mM
473	7-A11	ALX-400-040	Cisplatin (Cis-Diamineplatinum(Ii) Dichloride)	15663-27-1	300.1	10mM
474	7-B02	BML-AC2617	Cladribine	4291-63-8	285.7	10mM
475	7-B03	BML-AC3067	Clavulanate Potassium	61177-45-5	237.3	10mM
476	7-B04	BML-AC3068	Clobazam	22316-47-8	300.7	10mM
477	7-B05	BML-AC3069	Clofazimine	2030-63-9	473.4	10mM
478	7-B06	BML-AC572	Clomipramine·HCl	17321-77-6	351.3	10mM
479	7-B07	BML-AC3070	Clonazepam	1622-61-3	315.7	10mM
480	7-B08	BML-AC2327	Clotrimazole	23593-75-1	344.8	10mM
481	7-B09	BML-AC3071	Cloxacillin·Na	7081-44-9	457.9	10mM
482	7-B10	ALX-380-033	Colchicine	64-86-8	399.4	10mM
483	7-B11	BML-AC3072	Colistimethate·Na	8068-28-8	1634.9	10mM
484	7-C02	ALX-380-272	Colistin Sulfate	1264-72-8	1267.6	10mM
485	7-C03	BML-AC3073	Cortisone Acetate	50-04-4	402.5	10mM
486	7-C04	BML-AC3074	Cyclobenzaprine·HCl	6202-23-9	311.9	10mM
487	7-C05	BML-AC3075	Cyclopentolate	512-15-2	291.4	10mM
488	7-C06	BML-AC3076	Cycloserine	68-41-7	102.1	10mM
489	7-C07	BML-AC3077	Cysteamine·HCl	156-57-0	113.6	10mM
490	7-C08	BML-GR300	Dactinomycin (= Actinomycin D)	50-76-0	1255.4	10mM
491	7-C09	ALX-550-141	Dalfampridine (4-Aminopyridine)	504-24-5	94.1	10mM
492	7-C10	ALX-550-072	Dantrolene·Na	14663-23-1	314.3	10mM
493	7-C11	ALX-270-090	Dapsone	80-08-0	248.3	10mM
494	7-D02	BML-A201	Daptomycin	103060-53-3	1620.7	10mM
495	7-D03	BML-AC3079	Darifenacin·HBr	133099-07-7	426.6	10mM
496	7-D04	BML-AC3080	Darunavir	206361-99-1	547.7	10mM
497	7-D05	BML-AC3081	Dasatinib	302962-49-8	488	10mM
498	7-D06	BML-AC1526	Decitabine	2353-33-5	228.2	10mM
499	7-D07	BML-AC3082	Deferasirox	201530-41-8	373.4	10mM
500	7-D08	BML-R121	Deferoxamine Mesylate	138-14-7	656.8	10mM
501	7-D09	BML-AC3083	Demeclocycline·HCl	64-73-3	464.9	10mM
502	7-D10	BML-AR119	Desipramine·HCl	58-28-6	302.8	10mM
503	7-D11	BML-AC3084	Desogestrel	54024-22-5	310.5	10mM
504	7-E02	BML-AC3085	Desonide	638-94-8	416.5	10mM
505	7-E03	BML-AC3086	Desoximetasone	382-67-2	376.5	10mM
506	7-E04	BML-AC3087	Desvenlafaxine Succinate Hydrate	386750-22-7	263.4	10mM
507	7-E05	BML-AC3088	Dexchlorpheniramine Maleate	2438-32-6	390.9	10mM
508	7-E06	BML-AC3089	Dexmedetomidine·HCl	145108-58-3	236.7	10mM
509	7-E07		Blank			
510	7-E08	BML-AC3091	Dexrazoxane	24584-09-6	268.3	10mM
511	7-E09	BML-AC3092	Diatrizoate Meglumine	131-49-7	809.1	10mM
512	7-E10	BML-AC3093	Diazepam	439-14-5	284.7	10mM
513	7-E11	BML-AC3094	Dicloxacillin·Na Salt Monohydrate	13412-64-1	510.3	10mM
514	7-F02	BML-AC3095	Dicyclomine·HCl	67-92-5	309.5	10mM
515	7-F03	BML-AC3096	Dienogest	65928-58-7	311.4	10mM
516	7-F04		Blank			
517	7-F05	BML-AC3098	Difluprednate	23674-86-4	508.6	10mM
518	7-F06	BML-AC2000	Digoxin	20830-75-5	780.9	10mM
519	7-F07	BML-AC3099	Dimenhydrinate	523-87-5	470	10mM
520	7-F08	BML-AC2001	Disopyramide	3737/9/5	339.5	10mM
521	7-F09	BML-AC752	Dopamine·HCl	62-31-7	189.6	10mM
522	7-F10	BML-AC3100	Doripenem	148016-81-3	420.5	10mM
523	7-F11	BML-AC3101	Doxapram·HCl	7081-53-0	378.5	10mM
524	7-G02	BML-AC2010	Doxepin·HCl	1229-29-4	315.8	10mM
525	7-G03	BML-AC2008	Droperidol	548-73-2	379.4	10mM
526	7-G04	BML-AC3102	Drospirenone	67392-87-4	366.5	10mM
527	7-G05	BML-AC3103	Duloxetine·HCl	136434-34-9	333.9	10mM
528	7-G06	BML-AC3104	Dutasteride	164656-23-9	528.5	10mM

#	Plate Location	Cat No.	Name	CAS #	MW	Conc
529	7-G07	BML-AC3105	Dyphylline	479-18-5	254.2	10mM
530	7-G08	BML-AC2356	Econazole Nitrate	24169-02-6	444.7	10mM
531	7-G09		Blank			
532	7-G10	BML-AC3107	Eflornithine·HCl	68278-23-9	218.6	10mM
533	7-G11	BML-AC3108	Epinastine·HCl	108929-04-0	285.8	10mM
534	7-H02	BML-AC3109	Epirubicin·HCl	56390-09-1	580	10mM
535	7-H03	BML-AC3110	Eplerenone	107724-20-9	414.5	10mM
536	7-H04	BML-AC3111	Eptifibatide	188627-80-7	832	10mM
537	7-H05	ALX-380-274	Erythromycin	114-07-8	733.9	10mM
538	7-H06	BML-AC1524	Estramustine Phosphate·Na	52205-73-9	564.4	10mM
539	7-H07	BML-AC3112	Estropipate	7280-37-7	436.6	10mM
540	7-H08	BML-AC3113	Eszopiclone	138729-47-2	388.8	10mM
541	7-H09	BML-AC3114	Ethambutol Dihydrochloride	1070-11-7	277.2	10mM
542	7-H10		Blank			
543	7-H11	BML-AC2329	Ethinyl Estradiol	57-63-6	296.4	10mM
544	8-A02	BML-AC3116	Ethionamide	536-33-4	166.2	10mM
545	8-A03	BML-AC3117	Ethosuximide	77-67-8	141.2	10mM
546	8-A04	BML-AC3118	Etodolac	41340-25-4	287.4	10mM
547	8-A05	BML-AC3119	Etomidate	33125-97-2	244.3	10mM
548	8-A06	BML-AC3120	Etonogestrel	54048-10-1	324.5	10mM
549	8-A07	BML-AC3121	Everolimus	159351-69-6	958.2	10mM
550	8-A08	BML-AC2311	Ezetimibe	163222-33-1	409.4	10mM
551	8-A09	BML-AC3122	Febuxostat	144060-53-7	316.4	10mM
552	8-A10	BML-AC3123	Fexofenadine·HCl	153439-40-8	538.1	10mM
553	8-A11	BML-AC3124	Fingolimod	162359-55-9	343.9	10mM
554	8-B02	BML-AC3125	Flavoxate·HCl	3717-88-2	427.9	10mM
555	8-B03	BML-AC3126	Flucytosine	2022-85-7	129.1	10mM
556	8-B04	BML-AC2615	Fludarabine Phosphate	75607-67-9	365.2	10mM
557	8-B05	BML-AC3127	Fludrocortisone Acetate	514-36-3	422.5	10mM
558	8-B06	BML-AC3128	Flunisolide	3385-03 3	434.5	10mM
559	8-B07	BML-AC3129	Fluocinonide	356-12-7	494.5	10mM
560	8-B08	BML-AC3130	Fluorometholone	426-13-1	376.5	10mM
561	8-B09	BML-AC3131	Flurandrenolide	1524-88-5	436.5	10mM
562	8-B10		Blank			
563	8-B11	BML-AC3133	Fluticasone Propionate	80474-14-2	500.6	10mM
564	8-C02	BML-AC325	Fluvoxamine Maleate	61718-82-9	434.4	10mM
565	8-C03	BML-AC3134	Fomepizole	7554-65-6	82.1	10mM
566	8-C04	BML-AC3135	Formoterol	73573-87-2	344.4	10mM
567	8-C05	BML-AC3136	Foscarnet·Na (Sodium Phosphonoformate Tribasic Hexahydrate)	34156-56-4	300	10mM
568	8-C06	BML-AC3137	Fosfomycin Calcium	26016-98-8	176.1	10mM
569	8-C07	BML-AC3138	Fosphenytoin·Na Pentahydrate	92134-98-0	496.3	10mM
570	8-C08	BML-AC3139	Gemifloxacin	175463-14-6	389.4	10mM
571	8-C09	BML-AC3140	Glycopyrrolate Iodide	873295-32-0	445.3	10mM
572	8-C10	BML-NP119	Griseofulvin	126-07-8	352.8	10mM
573	8-C11	BML-AC3141	Guanidine·HCl	50-01-1	95.5	10mM
574	8-D02	BML-AC3142	Halcinonide	3093-35-4	455	10mM
575	8-D03	BML-AC3143	Halobetasol Propionate	66852-54-8	485	10mM
576	8-D04	BML-AC1549	Hexachlorophene	70-30-4	406.9	10mM
577	8-D05	BML-AC3144	Homatropine Methylbromide	80-49-9	370.3	10mM
578	8-D06	BML-AC2012	Hydralazine·HCl	304-20-1	196.6	10mM
579	8-D07	BML-AC3145	Hydrochlorothiazide	58-93-5	297.7	10mM
580	8-D08	BML-AC3146	Hydroflumethiazide	135-09-1	331.3	10mM
581	8-D09	BML-AC3147	Hydroxocobalamin·HCl	58288-50-9	1382.8	10mM
582	8-D10	BML-AC3148	Hydroxychloroquine Sulfate	747-36-4	434	10mM
583	8-D11	BML-AC3149	Hydroxyurea	127-07-1	76.1	10mM
584	8-E02	BML-AC1559	Hydroxyzine Dihydrochloride	2192-20-3	447.8	10mM
585	8-E03	BML-AC3150	Ibutilide Fumarate	122647-32-9	500.7	10mM
586	8-E04	BML-AC3151	Iloperidone	133454-47-4	426.5	10mM

#	Plate Location	Cat No.	Name	CAS #	MW	Conc
587	8-E05	BML-AC2017	Indinavir	150378-17-9	613.8	10mM
588	8-E06	BML-AC2613	Irbesartan	138402-11-6	428.5	10mM
589	8-E07	ALX-430-139	Irinotecan·HCl	100286-90-6	677.2	10mM
590	8-E08	BML-AC3152	Isocarboxazid	59-63-2	231.3	10mM
591	8-E09	ALX-400-008	Isosorbide Dinitrate	87-33-2	236.1	10mM
592	8-E10	BML-GR102	Isotretinoin (13-Cis-Retinoic Acid)	4759-48-2	300.4	10mM
593	8-E11	BML-CA213	Isradipine	75695-93-1	371.4	10mM
594	8-F02	ALX-380-049	Kanamycin Sulfate	25389-94-0	582.6	10mM
595	8-F03	BML-AC3153	Ketorolac Tromethamine	74103-07-4	376.4	10mM
596	8-F04	BML-AC2359	Labetalol·HCl	32780-64-6	364.9	10mM
597	8-F05	ALX-550-527	Lacosamide	175481-36-4	250.3	10mM
598	8-F06	BML-AC3154	Lactulose	4618-18-2	342.3	10mM
599	8-F07	BML-AC2301	Lamivudine	134678-17-4	229.3	10mM
600	8-F08	BML-AC3155	Lansoprazole	103577-45-3	369.4	10mM
601	8-F09	BML-AC2604	Lenalidomide	191732-72-6	259.3	10mM
602	8-F10	BML-AC3156	Leucovorin Calcium Pentahydrate	6035-45-6	601.6	10mM
603	8-F11	BML-AC3157	Levalbuterol·HCl	50293-90-8	275.8	10mM
604	8-G02	BML-AC3158	Levobunolol·HCl	27912-14-7	327.9	10mM
605	8-G03	BML-AC3159	Levocarnitine	541-15-1	161.2	10mM
606	8-G04	BML-AC3160	Levocetirizine Dihydrochloride	130018-87-0	461.8	10mM
607	8-G05	BML-AC3161	Levothyroxine·Na	55-03-8	798.9	10mM
608	8-G06	BML-AC2612	Lindane	58-89-9	290.8	10mM
609	8-G07	BML-AC3162	Liothyronine·Na	55-06-1	673	10mM
610	8-G08	BML-AC3163	Lopinavir	192725-17-0	628.8	10mM
611	8-G09	BML-AC3164	Lorazepam	846-49-1	321.2	10mM
612	8-G10	BML-AC3165	Loteprednol Etabonate	82034-46-6	467	10mM
613	8-G11	BML-AC1561	Loxapine Succinate	27833-64-3	445.9	10mM
614	8-H02	BML-AC3166	Mafenide·HCl	138-37-4	222.7	10mM
615	8-H03	BML-AC3167	Malathion	121-75-5	330.4	10mM
616	8-H04	BML-AC3168	Mannitol	69-65-8	182.2	10mM
617	8-H05	BML-AC3169	Maraviroc	376348-65-1	513.7	10mM
618	8-H06	BML-AC3170	Mechlorethamine·HCl	55-86-7	192.5	10mM
619	8-H07	BML-AC3171	Meclizine Dihydrochloride	1104-22-9	463.9	10mM
620	8-H08	BML-EI163	Meclofenamate·Na	6385-02-0	318.1	10mM
621	8-H09	BML-AC2020	Mefloquine·HCl	51773-92-3	414.8	10mM
622	8-H10	BML-AC3172	Mepenzolate Bromide	76-90-4	340.4	10mM
623	8-H11	BML-AC3173	Mepivacaine·HCl	1722-62-9	282.8	10mM
624	9-A02	BML-AC3174	Meprobamate (Schedule Iv)	57-53-4	218.3	10mM
625	9-A03	BML-AC3175	Mequinol	150-76-5	124.1	10mM
626	9-A04	BML-AC2360	Mercaptopurine Hydrate	6112-76-1	170.2	10mM
627	9-A05	BML-AC3176	Mesna	19767-45-4	164.2	10mM
628	9-A06	BML-AC3177	Mestranol	72-33-3	310.4	10mM
629	9-A07	BML-AC1569	Metaproterenol Hemisulfate (Orciprenaline)	5874-97-5	260.3	10mM
630	9-A08	BML-AC3178	Metaraminol Bitartrate	33402-03-8	317.3	10mM
631	9-A09	BML-AC3179	Metaxalone	1665-48-1	221.3	10mM
632	9-A10	BML-AC3180	Methacholine Chloride	62-51-1	195.7	10mM
633	9-A11	BML-AC3181	Methazolamide	554-57-4	236.3	10mM
634	9-B02	BML-AC3182	Methenamine Hippurate	5714-73-8	319.4	10mM
635	9-B03	BML-AC3183	Methocarbamol	532-03-6	241.2	10mM
636	9-B04	ALX-440-045	Methotrexate	133073-73-1	472.5	10mM
637	9-B05	BML-EI132	Methoxsalen (Xanthotoxin)	298-81-7	216.2	10mM
638	9-B06	BML-AC3184	Methscopolamine Bromide ((−)-Scopolamine Methyl Bromide)	155-41-9	398.3	10mM
639	9-B07	BML-AC3185	Methsuximide	77-41-8	203.2	10mM
640	9-B08	BML-AC3186	Methyclothiazide	135-07-9	360.2	10mM
641	9-B09	BML-AC3187	Methyl Aminolevulinate·HCl	79416-27-6	181.6	10mM
642	9-B10	BML-NP189	Methylergonovine Maleate	57432-61-8	455.5	10mM
643	9-B11	BML-AC2611	Metolazone	17560-51-9	365.8	10mM
644	9-C02	BML-EI256	Metyrapone	54-36-4	226.3	10mM

#	Plate Location	Cat No.	Name	CAS #	MW	Conc
645	9-C03		Blank			
646	9-C04	BML-AC2002	Mexiletine·HCl	5370-01 4	215.7	10mM
647	9-C05	BML-AC2328	Micafungin	235114-32-6	1270.3	10mM
648	9-C06	BML-AC2343	Miconazole	22916-47-8	416.1	10mM
649	9-C07		Blank			
650	9-C08	BML-AC3190	Midodrine·HCl	3092-17-9	254.9	10mM
651	9-C09	BML-AC3191	Miglitol	72432-03-2	207.2	10mM
652	9-C10	BML-AC2021	Milnacipran·HCl	101152-94-7	246.4	10mM
653	9-C11	BML-AC1366	Mirtazapine	61337-67-5	265.4	10mM
654	9-D02	BML-AC2341	Mitotane	53-19-0	320	10mM
655	9-D03	BML-AC1305	Modafinil (Schedule Iv)	68693-11-8	273.4	10mM
656	9-D04	BML-AC3192	Moexipril·HCl	82586-52-5	535	10mM
657	9-D05	BML-AC3193	Mometasone Furoate	83919-23-7	521.4	10mM
658	9-D06	BML-AC3194	Mupirocin	12650-69-0	500.6	10mM
659	9-D07	BML-AC3195	Nadolol	42200-33-9	309.4	10mM
660	9-D08	BML-AC3196	Nafcillin·Na	985-16-0	436.5	10mM
661	9-D09	BML-AC3197	Naftifine·HCl	65473-14-5	323.9	10mM
662	9-D10	BML-AC3198	Naratriptan·HCl	143388-64-1	371.9	10mM
663	9-D11	BML-AC2112	Natamycin	7681-93-8	665.7	10mM
664	9-E02	BML-AC3199	Nebivolol·HCl	152520-56-4	441.9	10mM
665	9-E03	BML-AC3200	Nelarabine	121032-29-9	297.3	10mM
666	9-E04	BML-AC3201	Nepafenac	78281-72-8	254.3	10mM
667	9-E05	BML-AC2306	Nevirapine	129618-40-2	266.9	10mM
668	9-E06	ALX-460-009	Niacin (Known As Vitamin B3, Nicotinic Acid And Vitamin Pp)	59-67-6	123.1	10mM
669	9-E07	BML-AC782	Nicotine	54-11-5	162.3	10mM
670	9-E08	BML-AC2005	Nilotinib	641571-10-0	529.5	10mM
671	9-E09	BML-AC3202	Nilutamide	63612-50-0	317.2	10mM
672	9-E10	BML-AC3203	Nitazoxanide	55981-09-4	307.3	10mM
673	9-E11	BML-AC3204	Nitisinone	104206-65-7	329.2	10mM
674	9-F02	BML-AC2317	Nitrofurantoin	67-20-9	238.2	10mM
675	9-F03	BML-AC2345	Nizatidine	76963-41-2	331.5	10mM
676	9-F04	BML-AC3205	Nortriptyline·HCl	894-71-3	299.8	10mM
677	9-F05	BML-AC3206	Olsalazine·Na	6054-98-4	302.2	10mM
678	9-F06	ALX-350-152	Orlistat (Tetrahydrolipstatin)	96829-58-2	495.7	10mM
679	9-F07	BML-AC3207	Oxaprozin	21256-18-8	293.3	10mM
680	9-F08	BML-AC3208	Oxazepam	604-75-1	286.7	10mM
681	9-F09	BML-AC3209	Oxtriphylline	4499-40-5	283.3	10mM
682	9-F10	BML-AC3210	Oxybutynin Chloride	1508-65-2	394	10mM
683	9-F11	BML-AC3211	Oxytetracycline·HCl	2058-46-0	496.9	10mM
684	9-G02	BML-AC2009	Paliperidone	144598-75-4	426.5	10mM
685	9-G03	BML-AC3212	Palonosetron·HCl	135729-62-3	332.9	10mM
686	9-G04	BML-AC3213	Paromomycin Sulfate	1263-89-4	713.7	10mM
687	9-G05	BML-AC3214	Pazopanib·HCl	635702-64-6	474	10mM
688	9-G06	BML-AC3215	Pemetrexed Disodium	150399-23-8	471.4	10mM
689	9-G07	BML-AC3216	Pemirolast Potassium	100299-08-9	266.3	10mM
690	9-G08	BML-AC3217	Penicillamine (D-Penicillamine)	52-67-5	149.2	10mM
691	9-G09	BML-AC2607	Penicillin G Potassium (Benzylpenicillin)	113-98-4	334.4	10mM
692	9-G10	BML-AC1801	Pentamidine Isethionate	140-64-7	592.7	10mM
693	9-G11	BML-AC2019	Pentostatin	53910-25-1	268.3	10mM
694	9-H02	BML-AC3218	Perindopril Erbumine	107133-36-8	441.6	10mM
695	9-H03	BML-PR103	Permethrin	52645-53-1	391.3	10mM
696	9-H04	BML-AC2349	Perphenazine	58-39-9	404	10mM
697	9-H05	BML-AC3219	Phenelzine Sulfate	156-51-4	234.3	10mM
698	9-H06	BML-AC3220	Phenylephrine	59-42-7	167.2	10mM
699	9-H07	BML-AC3221	Phytonadione	84-80-0	450.7	10mM
700	9-H08	BML-AC3222	Pimecrolimus	137071-32-0	810.5	10mM
701	9-H09	BML-AC3223	Pitavastatin Calcium	147526-32-7	881	10mM
702	9-H10		Blank			

#	Plate Location	Cat No.	Name	CAS #	MW	Conc
703	9-H11	BML-AC3225	Podofilox	518-28-5	414.4	10mM
704	10-A02	BML-AC3226	Posaconazole	171228-49-2	700.8	10mM
705	10-A03	BML-AC3227	Pralidoxime Chloride	51-15-0	172.6	10mM
706	10-A04	BML-AC3228	Prasugrel	150322-43-3	373.4	10mM
707	10-A05	BML-AC2309	Pravastatin·Na	81131-70-6	446.5	10mM
708	10-A06	BML-AC3229	Pregabalin	148553-50-8	159.2	10mM
709	10-A07	BML-AC3230	Prilocaine·HCl	1786-81-8	256.8	10mM
710	10-A08	BML-AC484	Primidone	125-33-7	218.3	10mM
711	10-A09	ALX-430-113	Probenecid	57-66-9	285.4	10mM
712	10-A10		Blank			
713	10-A11	BML-AC3232	Proparacaine·HCl	5875-06 9	330.9	10mM
714	10-B02	BML-AC2344	Propylthiouracil	51-52-5	170.2	10mM
715	10-B03	BML-AC3233	Protriptyline·HCl	1225-55-4	299.8	10mM
716	10-B04	BML-AC2314	Pyrazinamide	98-96-4	123.1	10mM
717	10-B05	BML-AC3234	Pyridostigmine Bromide	101-26-8	261.1	10mM
718	10-B06	BML-AC2358	Pyrimethamine	58-14-0	248.7	10mM
719	10-B07	BML-AC129	Quinidine·HCl·H2O	56-54-2	324.4	10mM
720	10-B08	BML-AC3235	Rabeprazole·Na	117976-90-6	381.4	10mM
721	10-B09	BML-AC3236	Raltegravir	871038-72-1	444.4	10mM
722	10-B10	BML-AC3237	Ramelteon	196597-26-9	259.3	10mM
723	10-B11	BML-AC3238	Rasagiline Mesylate	161735-79-1	267.3	10mM
724	10-C02	BML-AC3239	Regadenoson	313348-27-5	390.4	10mM
725	10-C03	BML-AC3240	Repaglinide	135062-02-1	452.6	10mM
726	10-C04	BML-NP254	Reserpine	50-55-5	608.7	10mM
727	10-C05	BML-AC3241	Rifabutin	72559-06-9	847	10mM
728	10-C06	BML-AC3242	Rifapentine	61379-65-5	877	10mM
729	10-C07	BML-AC3243	Rifaximin	80621-81-4	785.9	10mM
730	10-C08	BML-AC2014	Ritonavir	155213-67-5	721	10mM
731	10-C09	BML-AC2026	Rizatriptan Benzoate	145202-66-0	391.5	10mM
732	10-C10	BML-D113	Ropinirole·HCl	91374-20-8	296.8	10mM
733	10-C11	BML-AC3245	Ropivacaine·HCl Monohydrate	132112-35-7	274.4	10mM
734	10-D02	BML-AC2346	Rosuvastatin Calcium	147098-20-2	1001.1	10mM
735	10-D03	BML-AC3246	Rufinamide	106308-44-5	238.2	10mM
736	10-D04	BML-AC3247	Saquinavir Mesylate	149845-06-7	767	10mM
737	10-D05	BML-AC3248	Selegiline·HCl	14611-52-0	223.7	10mM
738	10-D06	BML-NS115	Sertraline·HCl	79559-97-0	342.7	10mM
739	10-D07	BML-AC3249	Silver Sulfadiazine	22199-08-2	357.1	10mM
740	10-D08	BML-AC3251	Sitagliptin Phosphate	654671-77-9	505.3	10mM
741	10-D09	BML-AC1514	Sorafenib Tosylate	475207-59-1	637	10mM
742	10-D10	BML-AC2302	Stavudine	3056-17-5	224.2	10mM
743	10-D11	ALX-380-010	Streptozocin	18883-66-4	265.2	10mM
744	10-E02	BML-AC3252	Sulconazole Nitrate	61318-91-0	460.8	10mM
745	10-E03	BML-AC3253	Sulfacetamide·Na	127-56-0	236.2	10mM
746	10-E04	BML-AC2319	Sulfamethoxazole	723-46-6	253.3	10mM
747	10-E05	BML-AC3254	Sulfanilamide	63-74-1	172.2	10mM
748	10-E06	BML-AC2013	Sunitinib Malate	341031-54-7	532.6	10mM
749	10-E07	ALX-380-008	Tacrolimus (Fk506)	104987-11-3	804	10mM
750	10-E08	BML-AC3255	Tadalafil	171596-29-5	389.4	10mM
751	10-E09	BML-AC3256	Tazarotene	118292-40-3	351.5	10mM
752	10-E10	BML-AC3257	Telbivudine	3424-98-4	242.2	10mM
753	10-E11	BML-AC3258	Telithromycin	191114-48-4	812	10mM
754	10-F02	BML-AC3259	Temazepam	846-50-4	300.7	10mM
755	10-F03	BML-AC3260	Temsirolimus	162635-04-3	1030.3	10mM
756	10-F04	ALX-400-053	Teniposide	29767-20-2	656.7	10mM
757	10-F05	BML-AC2303	Tenofovir	147127-20-6	287.2	10mM
758	10-F06	BML-AC3261	Terbutaline Hemisulfate	23031-32-5	274.3	10mM
759	10-F07	BML-AC3262	Terconazole	67915-31-5	532.5	10mM
760	10-F08	BML-AC3263	Testosterone Enanthate	315-37-7	400.6	10mM
761	10-F09	BML-AC3264	Tetrabenazine	58-46-8	371.4	10mM

#	Plate Location	Cat No.	Name	CAS #	MW	Conc
762	10-F10	BML-AC3265	Tetrahydrozoline·HCl	522-48-5	236.7	10mM
763	10-F11	ALX-480-062	Theophylline	58-55-9	180.2	10mM
764	10-G02	BML-AC1532	Thioguanine (6-Thioguanine)	154-42-7	167.2	10mM
765	10-G03	BML-AC2601	Thiotepa	52-24-4	189.2	10mM
766	10-G04		Blank			
767	10-G05	BML-AC3267	Tiagabine·HCl	145821-59-6	375.6	10mM
768	10-G06	BML-AC3268	Tigecycline	220620-09-7	585.7	10mM
769	10-G07	BML-AC3269	Tiludronate Disodium	149845-07-8	362.6	10mM
770	10-G08	BML-AC3270	Tiopronin	1953-02 2	163.2	10mM
771	10-G09	BML-AC3271	Tirofiban·HCl	150915-40-5	477.1	10mM
772	10-G10	BML-AC3272	Tolterodine Tartrate	124937-52-6	475.6	10mM
773	10-G11	BML-AC3273	Tolvaptan	150683-30-0	448.9	10mM
774	10-H02	BML-AC3274	Topiramate	97240-79-4	339.4	10mM
775	10-H03	BML-AC3275	Torsemide	56211-40-6	348.4	10mM
776	10-H04	ALX-270-493	Trandolapril	87679-37-6	430.5	10mM
777	10-H05	BML-AC3276	Travoprost	157283-68-6	500.6	10mM
778	10-H06	BML-AC2318	Trazodone·HCl	25332-39-2	408.3	10mM
779	10-H07	BML-AC2357	Tretinoin	302-79-4	300.4	10mM
780	10-H08	BML-AC3277	Triamcinolone Acetonide	76-25-5	434.5	10mM
781	10-H09	ALX-440-043	Triamterene	396-01-0	253.3	10mM
782	10-H10	BML-AC3278	Triazolam	28911-01-5	343.2	10mM
783	10-H11	BML-AC3279	Trientine Dihydrochloride	38260-01-4	219.2	10mM
784	11-A02	BML-AC3280	Trihexyphenidyl·HCl	52-49-3	337.9	10mM
785	11-A03	BML-AC3281	Trimethadione	127-48-0	143.1	10mM
786	11-A04	BML-AC3282	Trimethobenzamide·HCl	554-92-7	424.9	10mM
787	11-A05	BML-AC3283	Trimipramine Maleate	521-78-8	410.5	10mM
788	11-A06	BML-AC3284	Trospium Chloride	10405-02-4	428	10mM
789	11-A07	BML-AC3285	Ursodiol	128-13-2	392.6	10mM
790	11-A08	BML-AC3286	Valganciclovir·HCl	175865-59-5	390.8	10mM
791	11-A09	ALX-550-304	Valproate·Na	1069-66-5	166.2	10mM
792	11-A10	BML-AC3287	Valsartan	137862-53-4	435.5	10mM
793	11-A11	ALX-380-279	Vancomycin·HCl	1404-93-9	1485.7	10mM
794	11-B02	BML-AC3288	Varenicline Tartrate	375815-87-5	361.4	10mM
795	11-B03	BML-AC506	Vigabatrin	60643-86-9	129.2	10mM
796	11-B04	BML-AC3289	Voriconazole	137234-62-9	349.3	10mM
797	11-B05	BML-AC2354	Warfarin·Na	129-06-6	330.3	10mM
798	11-B06	BML-AC3290	Zaleplon	151319-34-5	305.3	10mM
799	11-B07	BML-AC3291	Zanamivir	139110-80-8	332.3	10mM
800	11-B08	BML-AC1570	Ziprasidone	122883-93-6	412.9	10mM
